# In Vitro Antitumor Effects of Melittin Attached to Fe_3_O_4_ Magnetic Nanoparticles with Synergistic Contribution of Magnetic Hyperthermia

**DOI:** 10.3390/molecules31122171

**Published:** 2026-06-20

**Authors:** Alex Câmpian, Ioana Bâldea, Mara Muntean, Cristian Iacoviță, Adrian Florea

**Affiliations:** 1 Department of Cell and Molecular Biology, Faculty of Medicine, “Iuliu Hațieganu” University of Medicine and Pharmacy, 6 Louis Pasteur St., 400349 Cluj-Napoca, Romania; alexcampian@yahoo.com (A.C.); muntean.mara@elearn.umfcluj.ro (M.M.); aflorea@umfcluj.ro (A.F.); 2 Department of Physiology, Faculty of Medicine, “Iuliu Hațieganu” University of Medicine and Pharmacy, 1–3 Clinicilor St., 400012 Cluj-Napoca, Romania; ioana.baldea@umfcluj.ro; 3 Department of Pharmaceutical Physics-Biophysics, Faculty of Pharmacy, “Iuliu Hațieganu” University of Medicine and Pharmacy, 6 Louis Pasteur St., 400349 Cluj-Napoca, Romania

**Keywords:** melittin-functionalized magnetic nanoparticles, Caco-2 cells, BJ fibroblasts, magnetic hyperthermia, apoptosis, mitochondria, transmission electron microscopy, antitumor activity

## Abstract

Melittin (Mel) is a membrane-active peptide with potential anticancer activity, but its direct therapeutic application may be limited by nonspecific toxicity and delivery-related challenges. The study aimed to assess melittin-functionalized magnetic nanoparticles (MNPs-Mel) as a strategy to enhance antitumor activity in Caco-2 cells, with/without magnetic hyperthermia (MH) association. BJ fibroblasts were used as a normal human in vitro cellular model. The effects of free Mel (2.5 µg/mL), MNPs, and MNPs-Mel (50 µg/mL both) + MH (30 min at 355 kHz and 25 kA/m) were assessed using colorimetry (for viability), luminescence (ATP), and spectrophotometry (lactate) following different exposure conditions. The mechanism of apoptosis induction was evaluated by ELISA (caspase 8 and 9 levels). Transmission electron microscopy (TEM) was also used to evaluate nanoparticle morphology and treatment-associated cellular ultrastructural changes. Free Mel reduced viability in both cell lines, with Caco-2 cells showing greater sensitivity at lower concentrations. MNPs (with/without MH) produced limited and less consistent effects, whereas MNPs-Mel significantly reduced Caco-2 viability and ATP levels and increased LDH and caspase 9. MH further enhanced the effects of MNPs-Mel: reduced viability (57–58% of the control at 24 h and 72 h), decreased ATP levels (67% of the control at 24 h and 53% at 72 h), increased LDH levels (206% of the control at 24 h and 301% at 72 h), and induced the mitochondrial apoptotic pathway (caspase 9 increased with 2164% of the control at 72 h). TEM proved the internalization of both MNPs and MNPs-Mel and revealed extensive ultrastructural alterations concerning mitochondria and lysosomes produced by MNPs-Mel, particularly in the Caco-2 cells. These modifications were heavily increased by MNPs-Mel + MH exposure. Overall, these findings demonstrate that Mel functionalization increases the antitumor activity of Mel at lower doses and that MH further potentiates this effect in Caco-2 cells.

## 1. Introduction

### 1.1. Cancer and the Caco-2 In Vitro Model

Cancer remains a leading global health burden, with colorectal cancer (CRC) being one of the most diagnosed and deadly malignancies, underscoring the urgent need for new therapies and improved preclinical models due to its biological complexity and treatment resistance. Current CRC treatment (for stage III disease) relies on chemotherapy, where fluoropyrimidine-based regimens combined with oxaliplatin have shown improved disease-free and overall survival [1,2,3,4]. Depending on the CRC type, several antibodies (anti-EGFR, anti-VEGF, [5,6]) or immune checkpoint inhibitors [7,8] also demonstrated superior progression-free survival compared with chemotherapy in the first-line setting. CRC’s heterogeneity—marked by genetic, epigenetic, and signaling alterations—makes in vitro models essential for evaluating anticancer agents, as they allow direct assessment of cellular responses like viability, proliferation, and apoptosis, despite their limitations in replicating the tumor microenvironment [9,10,11]. The Caco-2 cell line, derived from human colorectal adenocarcinoma, is a widely used in vitro model for intestinal epithelial studies, transport, barrier function, and cancer research due to its epithelial differentiation and stability. Despite several limitations discussed below, it remains highly useful for assessing the antitumor activity of novel nanosystems under controlled conditions [12,13,14].

### 1.2. Melittin as a Bioactive Anticancer Peptide

In the context of systemic side effects of chemotherapy, new approaches like the potential use of natural, bioactive molecules are being explored for their antitumor potential. Melittin (Mel) is a 26-amino-acid, amphipathic peptide accounting for 50–55% of dry honeybee venom [15,16], which is mostly responsible for its biological activity and toxicity. Its ability to alter membrane structure and permeability in a concentration-dependent manner makes it a useful tool in both cancer and drug-delivery research [17,18,19,20,21,22]. But Mel’s anticancer potential stems from diverse mechanisms (membrane disruption, mitochondrial dysfunction, oxidative stress, apoptosis induction, and interference with proliferation and metastasis) observed across various cancer models, indicating broad antitumor activity rather than specificity to a single tumor type. Given the global significance of CRC and the need for new therapies, the distinct properties of Mel set it apart from conventional chemotherapeutics, making it a promising candidate for novel anticancer platforms, especially in drug-resistant settings [17,22,23,24,25,26].

Despite its antitumor potential, the therapeutic use of free Mel is limited by poor selectivity, significant cytotoxicity toward non-malignant cells, instability in biological environments, and uncontrolled distribution, leading to systemic toxicity [17,18,23,26]. However, tumor cells are highly sensitive and do not develop resistance to Mel. To address these challenges, recent research has focused on nanocarrier-based delivery systems, which improve stability, modulate membrane interaction, reduce premature degradation, and enhance selectivity, overcoming barriers like hemolytic behavior [17,26,27,28]. Additionally, Mel-functionalized nanomaterials enable the use of lower effective concentrations (reviewed in [22]). Melittin-based nanotherapy extends beyond simple encapsulation, as Mel can be covalently conjugated, adsorbed, electrostatically associated, or integrated into multifunctional nanostructures to enhance delivery and enable combination with targeting or imaging strategies. This flexibility allows Mel to be part of a broader therapeutic architecture, transforming it from a reactive free peptide into a programmable component of controlled therapeutic systems [26,27,28].

### 1.3. Magnetic Nanoparticles

Magnetic nanoparticles (MNPs) combine nanoscale physicochemical properties with magnetic responsiveness, enabling applications like magnetic resonance imaging, magnetic targeting, biosensing, separation, cell tracking, and magnetic hyperthermia (MH) by leveraging external magnetic fields for localization, detection, and therapy. Among these, iron oxide nanoparticles—especially magnetite (Fe_3_O_4_)—are the most studied due to their strong magnetism, adaptable surface chemistry, and biocompatibility, supported by extensive synthesis and functionalization research [29,30,31,32,33]. MNP platforms are ideal for Mel functionalization, as they offer a stable scaffold for peptide attachment while allowing surface engineering to enhance dispersibility, preserve bioactivity, and minimize uncontrolled biological interactions by magnetic guidance—benefits that are critical for mitigating Mel’s systemic toxicity [22]. The value of such systems lies in combining peptide bioactivity with the structural and functional benefits of magnetic nanoplatforms [26,27,28].

In the current study, attaching Mel to Fe_3_O_4_ MNPs offers a controlled framework for delivering Mel to the Caco-2 cells as a suitable in vitro model. A first objective was to investigate the molecular and ultrastructural effects of low doses of Mel delivered intracellularly by melittin-functionalized MNPs (MNPs-Mel) in comparison with those triggered by similar doses of free Mel. A second objective was to establish the contribution of alternating magnetic field (AMF) exposure to the antitumor effect of MNPs-Mel, and to assess whether this coupling enhances anticancer efficacy.

## 2. Results and Discussions

### 2.1. Melittin-Coated MNPs

**Figure 1:**
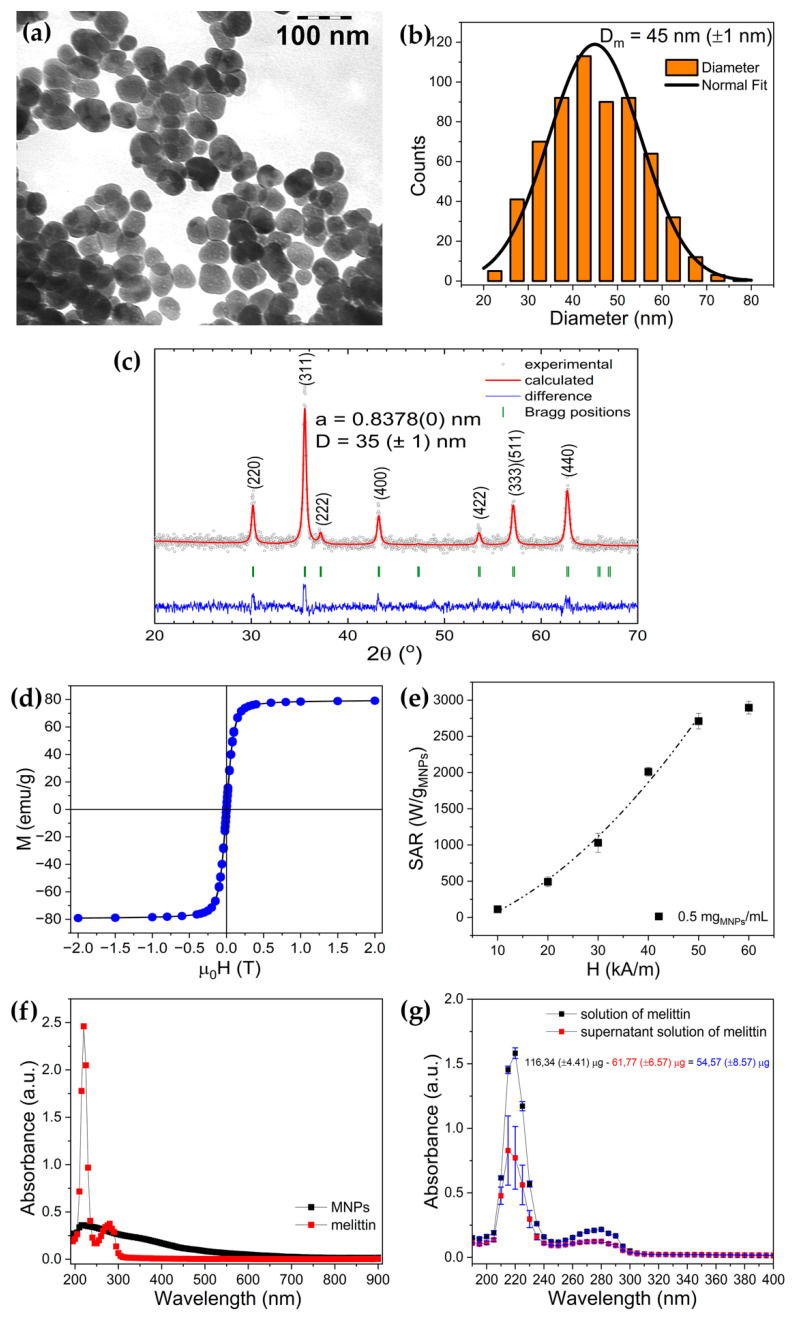
(**a**) Relevant TEM image of MNPs. (**b**) Size distribution histograms of MNPs fitted to a normal distribution. (**c**) XRD diffraction pattern and Rietveld refinement of MNPs. (**d**) Hysteresis loops of MNPs at 300 K. (**e**) SAR values of MNPs dispersed in water at a concentration of 500 µg MNPs/mL, with the dash-dot-dot line indicating a parabolic fit of SAR as a function of AMF amplitude (*H*). (**f**) UV-Vis absorption spectra of aqueous solutions containing either MNPs (10 µg/mL) or Mel (1250 µg/mL). (**g**) UV-Vis spectra of aqueous solutions of Mel and supernatant solutions of Mel, both representing the average of 16 individual measurements.

Through the thermal decomposition of an iron acetylacetonate precursor in a high-boiling-point solvent (dibenzyl ether), and in the presence of oleic acid and ethylene glycol, elongated spherical MNPs were synthesized. These MNPs exhibited an average diameter of 45 nm, as shown in Figure 1a,b. The diffractogram (Figure 1c) confirmed the formation of a pure inverse spinel crystalline structure in the MNPs, which is consistent with magnetite (Fe_3_O_4_) as referenced in the PDF database (PDF number: 88-0315). The measured lattice parameter (a = 8.378 Å) closely matched that of bulk magnetite (a = 8.375 Å). Using Scherrer’s formula, the average crystallite size was calculated from the (220), (311), and (440) diffraction peaks, yielding a value of 35 nm. This size is approximately 10 nm smaller than the average diameter observed in TEM images, suggesting that some MNPs may possess a double-core structure. The hysteresis loops recorded at 300 K (Figure 1d) displayed negligible remanence and coercivity, indicating a superparamagnetic behavior of MNPs at room temperature. The saturation magnetization (M_s_) for the elongated spherical MNPs is 80 emu/g, which is slightly lower than that of bulk magnetite (92 emu/g), further supporting their identification as pure magnetite. The specific absorption rate (SAR) of the MNPs increased from 110 W/g at a magnetic field amplitude (*H*) of 10 kA/m to a value of 2900 W/g at the highest tested amplitude of 60 kA/m (Figure 1e). Notably, between 10 and 50 kA/m, the SAR follows a quadratic relationship with *H*, which is consistent with the superparamagnetic behavior of the MNPs [34]. Beyond 50 kA/m, however, a saturation effect becomes evident (Figure 1e).

For H ranging from 10 to 30 kA/m—values that comply with the biological safety limit (*H*·*f* = 9.59 × 10^−9^ A·m^−1^·s^−1^) [35]—the observed SAR values are significantly higher than those reported for most spherical MNPs in the literature [30]. This makes these MNPs highly suitable candidates for in vitro MH applications.

The UV-Vis absorption spectrum of the MNPs displayed strong absorption in the UV region (200–400 nm), with a gradual decrease in intensity extending into the visible region (400–900 nm) (Figure 1f). Mel exhibited two distinct absorption peaks at 220 nm and 280 nm within the UV range, with no detectable absorption in the visible spectrum (Figure 1f) [36]. The peak at 280 nm was utilized to quantify the amount of Mel adsorbed onto the MNPs, which was determined using a calibration curve detailed in the Supplementary Materials (Section SI2). Following the removal of MNPs-Mel, the UV-Vis spectra of the remaining solution revealed a reduced absorbance at 280 nm compared to the initial Mel solution (Figure 1g), indicating that 61.77 (±6.57) μg of Mel remained unadsorbed. By subtraction, it was determined that 54.57 (±8.57) μg of Mel was successfully adsorbed, resulting in a concentration of 54.57 µg Mel/mg MNPs (Figure 1g).

In peptide-based systems, surface coating of MNPs is particularly relevant because peptides in free form often show limited stability, rapid degradation, or poor selectivity. In such cases, the nanoparticle surface acts not only as support for loading, but also as a means of modulating the behavior of the attached biomolecule [32,37,38,39]. In MH, iron oxide nanoparticles convert electromagnetic energy into heat, potentially inducing tumor cell stress, apoptosis, ferroptosis, or increased sensitivity to chemotherapy, while limiting damage to surrounding tissues when heating is properly localized [30,40]. However, their effectiveness and safety depend on nanoparticle size, coating, aggregation state, surface functionalization, heating efficiency, concentration, biodistribution, and tumor selectivity [30]. In this context, functionalization with bioactive molecules such as Mel may further increase the antitumoral potential of magnetic nanoparticles by combining membrane-active biological effects with MH. Thus, Mel-functionalized MNPs represent a rational extension of previously reported Fe_3_O_4_-based cancer nanoplatforms, particularly for CRC models such as Caco-2 cells. On the other hand, a nanoparticle platform may improve the formulation stability of Mel, enhance cellular internalization, enable controlled attachment, and create a system that is more versatile than the free peptide alone, acting thus as an active therapeutic enhancer. Within such a design, apart from representing only a carrier core, Fe_3_O_4_ is a structural element that may support future imaging, magnetic targeting, or combination therapy strategies [32,38,39,41]. For an in vitro study performed in Caco-2 colon cancer cells, the use of melittin-functionalized Fe_3_O_4_ nanoparticles can therefore be justified on multiple levels. Their superparamagnetic behavior supports possible future theranostic development, and surface engineering enables biomolecule immobilization while preserving formulation flexibility. This makes MNPs a rational material platform for investigating whether peptide functionalization can be combined with a biologically relevant magnetic nanosystem in an anticancer setting [32,33,38,42,43].

The literature data shows that MNP surfaces can be functionalized with drugs, polymers, peptides, antibodies, or targeting ligands, allowing them to improve local delivery and reduce systemic exposure. Several studies support the relevance of MNPs in cancer therapy, including CRC models. Ren et al. used multifunctional Fe_3_O_4_ nanoparticles loaded with daunorubicin and 5-bromotetrandrine in combination with MH, showing that this approach could help overcome multidrug resistance [40]. In the same line, Chen et al. reported that Fe_3_O_4_ combined with gambogic acid affected the PI3K/Akt/Bad pathway in LOVO colon cancer cells [44], while Genc et al. showed that Fe_3_O_4_ nanoparticles combined with 5-fluorouracil exerted stronger antitumoral effects than 5-fluorouracil alone in a Caco-2 colon cancer model, including at lower drug concentrations [45]. Other Fe_3_O_4_-based nanoplatforms have also been investigated in CRC, including PPy@Fe_3_O_4_ nanoparticles, which were reported to inhibit proliferation and metastasis through suppression of NF-κB signaling and promotion of ferroptosis [46], as well as gambogic acid-containing Fe_3_O_4_ nanoparticles targeting SLC7A11 [47].

Recent studies further demonstrate the versatility of Fe_3_O_4_ nanoparticles as drug delivery and antitumoral platforms. Curcumin-loaded PLA-HA/Fe_3_O_4_ MNPs were evaluated in HCT116 CRC cells and were reported to show reduced toxicity toward non-cancerous cells [48]. Fe_3_O_4_ nanoparticles capped with silver were shown to induce apoptosis in colon cancer cells through increased reactive oxygen species and DNA damage [49]. Other recent approaches include pH-responsive chitosan-modified Fe_3_O_4_ nanoparticles loaded with β-acids for CRC treatment [50] and pegylated Fe_3_O_4_-CAPE MNPs designed for targeted therapy and MH treatment of CRC [51]. Together, these studies highlight the adaptability of MNPs for drug delivery, MH, oxidative stress induction, ferroptosis modulation, and pathway-targeted antitumoral strategies.

### 2.2. Cell Viability

**Figure 2:**
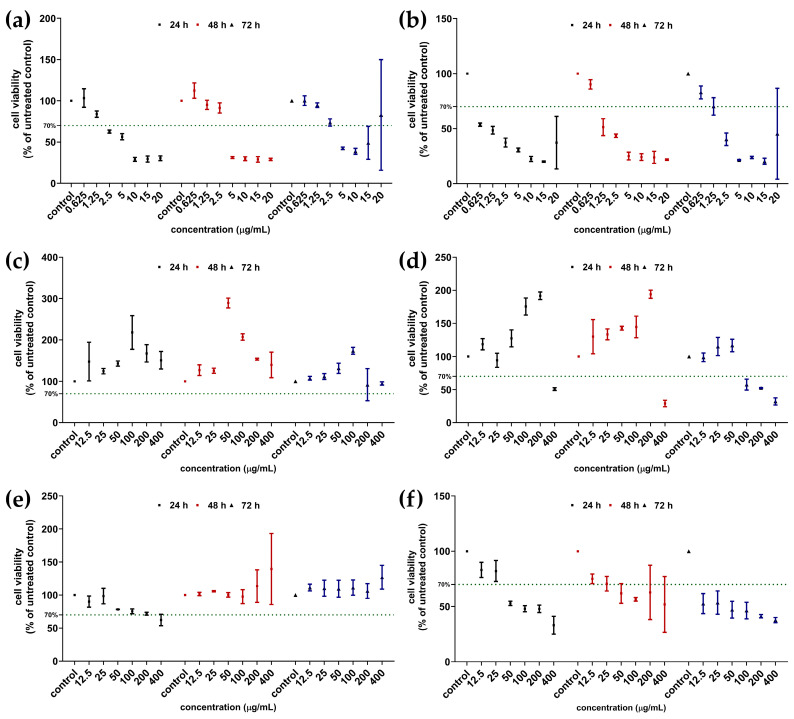
Viability of BJ cells (left panels) and Caco-2 cells (right panels) exposed to various concentrations of melittin (**a**,**b**), MNPs (**c**,**d**), and MNPs-Mel (**e**,**f**) at three different time points (24 h, 48 h, and 72 h). Data (*n* = 3) are presented as % of the untreated controls (mean ± SD); the line at 70% represents the limit for toxic effects.

Melittin showed a strong toxicity towards fibroblasts, with viability decreasing to ~63% of the control at doses of 2.5 µg/mL at 24 h, while 10 µg/mL reduced it to ~29% of the control. This effect was diminished with longer exposures, with the toxicity level being increased to 5 µg/mL after 48 and 72 h, possibly because of Mel denaturation and metabolic inactivation of the molecule by the cells (Figure 2a). Melittin toxicity was also increased in Caco-2 compared to normal cells, being induced at the minimal used concentration 0.625 µg/mL at 24 h (~54% of the control), 1.25 µg/mL at 48 h, and 2.5 µg/mL at 72 h (Figure 2b). The same trend of Mel toxicity diminishing with longer exposure times was observed in the case of Caco-2 as in the BJ cultures.

BJ cell viability was not significantly modified by exposure to MNPs (Figure 2c), with no toxicity up to 400 µg/mL. In the case of MNPs-Mel, higher concentrations (≥200 µg/mL) decreased viability in shorter exposures (24 h) to 71.67% of the control (200 µg/mL) and to 62.16% of the control (400 µg/mL), but the viability fully recovered after longer exposures (Figure 2e). In the Caco-2 cells, MNPs showed toxicity only at high concentrations (400 µg/mL) at 24 h (50.8% of the control) and at 48 h (28% of the control). The longer exposure time had an opposite effect to the one exhibited in BJ cultures, leading to an increased toxicity at 72 h from 100 µg/mL upward (57.8, 52.4, and 32.3% of the control) (Figure 2d). MNPs-Mel showed increased toxicity towards Caco-2 at all time points proportional to the exposure time. Thus, the dose of 50 µg/mL reduced the viability of cancer cells by 67.3% at 24 h, 64.8% at 48 h, and 43.1% at 72 h compared to the normal cells (Figure 2f).

### 2.3. Magnetic Hyperthermia Exposure

Magnetic nanoparticles possess the intrinsic property to dissipate energy when exposed to an AMF, including as heat, resulting in a rise in local temperature of several degrees, which is enough to disturb the metabolic pathways and trigger apoptosis in the cancer cells, which are generally known to be more heat-sensitive than normal cells. Testing of MNPs in our study design produced values close to the control, and showed that MH alone had a limited effect. It was demonstrated that this effect depends on the MNP concentration and functionalization. Torres-Lugo et al. reported significant cytotoxic differences generated by MH in Caco-2 and MCF-7 cells incubated with high concentrations of MNPs functionalized with carboxymethyl dextran (that improved particle dispersion in the culture medium) [52]. MNP functionalization with poly[3,4-dihydroxybenzhydrazide] (used as stabilizer) enhanced selective antitumor efficacy and induced cell death in Caco-2 and A375 cells at a higher rate than in normal BJ fibroblasts [53].

**Figure 3:**
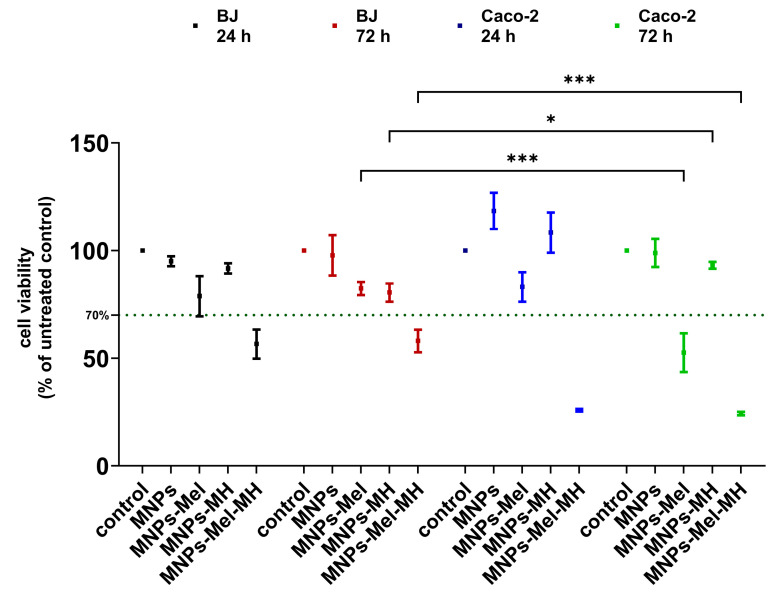
Viability of BJ cells (left) and Caco-2 cells (right) incubated for 24 h with 50 µg/mL MNPs or MNPs-Mel with or without exposure to 30 min MH at 25 kA/m at 24 h and 72 h. Data (n = 3) are presented as the % of the untreated controls (mean ± SD); the line at 70% represents the limit for toxic effects. * = *p* < 0.05, and *** = *p* < 0.0001.

In the current study, the MH effect was additive to that of Mel. However, there is no relationship between the intensity of the AMF and the decrease in cell viability (Figure 3). The most important factor that leads to the antitumor toxicity was related to the MH-mediated increased intracellular delivery of Mel by MH, leading to such toxic effects. Moreover, the Brownian movement generated under the influence of the AMF could be responsible for the release of Mel inside the endosomes, lysosomes, or cytosol. This assumption is also supported by the low temperature increase (39–40 °C) obtained by using only a low dose of MNPs-Mel (50 µg/mL). An interesting observation is a lack of a rebound effect on the viability obtained with MNPs-Mel and Mel at 72 h, which demonstrates a higher antitumor efficacy of the combined exposure. Based on these data, the MNPs-Mel dose of 50 µg/mL and the intensity of MH of 25 kA/m were selected for further experimental measurements. Both cell lines showed decreased viability at both time points when exposed to MNPs-Mel-MH (Figure 3).

In fibroblasts, the effect was less important in the 72 h exposure, with some viability recuperation compared to the 24 h exposure (51.5% of the control). On the contrary, in Caco-2 cells, at 25 kA/m, MNPs-Mel-MH treatment reduced absorbance to 26.9% of the control at 24 h and to 24.3% at 72 h, suggesting that small changes in the experimental protocol, such as a further increase in Mel dose by 15–20% or a longer exposure of cells to the AMF, could irreversibly damage all the cancer cells.

### 2.4. ATP

**Figure 4:**
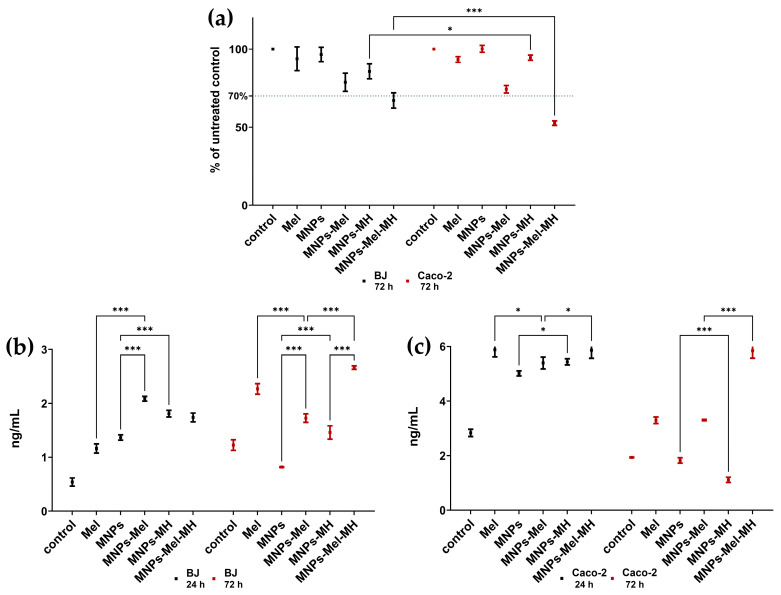
ATP (**a**) and LDH (**b**,**c**) levels in BJ cells and Caco-2 cells incubated for 24 h with 2.5 µg/mL melittin and with 50 µg/mL MNPs or MNPs-Mel, with or without exposure for 30 min to MH at 25 kA/m assessed at 72 h for ATP (n = 4), and at two different time points (24 h and 72 h) for LDH (n = 3). ATP data are presented as the % of the untreated controls and concentration values for LDH (mean ± SD). The dotted line at 70% represents the limit for toxic effects. * = *p* < 0.05, and *** = *p* < 0.0001.

The ATP assay was used to evaluate changes in cellular energetic/metabolic status following treatment. Cellular production of ATP (Figure 4a) following therapy was decreased in both cell lines in the Mel exposure and MNPs-Mel with or without MH. Because the absolute ATP control values differed between BJ and Caco-2 cells, the results were normalized and expressed as a percentage of the untreated control for each cell line. This normalization allowed comparison of the treatment-induced ATP reduction relative to the basal metabolic activity of each cell type, rather than comparing raw ATP values that may reflect intrinsic differences between cell lines. At 72 h, Sidak’s multiple comparisons test showed no significant differences in BJ versus Caco-2 for the untreated control, Mel, MNPs, or MNPs-Mel groups, suggesting that these treatments produced broadly comparable relative effects in both cell lines. However, after MNPs-MH exposure, ATP was significantly lower in BJ cells compared with Caco-2 cells (85.8% vs. 94.5%, adjusted *p* = 0.02), indicating a stronger relative metabolic effect of this treatment in BJ cells. In contrast, in the MNPs-Mel-MH group, the most pronounced ATP reduction was noted, especially in Caco-2 cells, where ATP decreased to 52.7%, compared with 67.1% in BJ cells (adjusted *p* < 0.001). Therefore, although some treatments affected both cell lines similarly, the triple combination showed a selective reduction in ATP in Caco-2 cells, supporting a greater cytotoxic or metabolic inhibitory effect of the combined MNPs, Mel, and MH treatment on the cancer cell line. No interactions of Mel with inner mitochondrial membrane proteins have been demonstrated to date; therefore, the reduction in ATP synthesis could be attributed to the peptide-mediated destabilization of this membrane, which is a supposition sustained by our TEM observations (see Section 2.7.6).

Our findings are in line with the reactions of tumor cells previously described by other researchers. Thus, Alonezi et al. reported ATP and metabolic changes produced by Mel in cisplatin-sensitive and resistant ovarian cancer cells [54]. The same group showed reduced ATP levels and altered energy metabolism in ovarian cancer cells consecutive to Mel + cisplatin incubation of ovarian cancer cells [55]. Other studies indicated the lower ATP levels as a marker in immunogenic cell death under the influence of D-melittin polymeric nanoparticles [56] or in treatments with oncolytic virus combined with CpG-Mel [57].

### 2.5. Lactate

Lactate assay was used for the assessment of cellular metabolism and loss of membrane integrity, leading to the release of lactate and LDH into the surrounding medium. Increased LDH levels are therefore commonly used to estimate cellular injury, necrotic/late apoptotic damage, or reduced cellular tolerance to treatment. In this context, LDH analysis was used to determine whether the tested formulations induced direct cytotoxic effects in BJ and Caco-2 cells after 24 h and 72 h (Figure 4b,c).

A three-way ANOVA revealed that LDH levels were significantly influenced by the experimental model. The strongest source of variation was cell type, accounting for 46.9% of total variation, F(1, 48) = 6162, *p* < 0.001, indicating a marked difference in LDH response between BJ and Caco-2 cells.

Treatment also had a significant effect, accounting for 18.9% of total variation, F(5, 48) = 497, *p* < 0.001, while time accounted for 7.61%, F(1, 48) = 1001, *p* < 0.001. Significant interaction effects were observed for treatment × cell type, F(5, 48) = 112, *p* < 0.001; treatment × time, F(5, 48) = 198, *p* < 0.001; cell type × time, F(1, 48) = 1566, *p* < 0.001; and treatment × cell type × time, F(5, 48) = 65.9, *p* < 0.001. These results show that LDH release was not determined by treatment alone, but by the combined influence of cell type, treatment composition, and exposure duration. The two cell lines showed clearly distinct LDH profiles. In BJ cells, LDH levels were comparatively low and increased gradually with treatment and time. Two-way ANOVA confirmed significant effects of treatment, F(5, 24) = 255, *p* < 0.001; time, F(1, 24) = 91.2, *p* < 0.001; and treatment × time interaction, F(5, 24) = 141, *p* < 0.001. Treatment was the dominant source of variation in BJ cells, explaining 60.8% of total variation, while the interaction between treatment and time explained 33.7%. This suggests that BJ cytotoxicity was mainly determined by the type of treatment, but that the magnitude and ranking of the response shifted over time.

In contrast, Caco-2 cells displayed a much stronger early LDH response. Two-way ANOVA showed significant effects of treatment, F(5, 24) = 315, *p* < 0.001; time, F(1, 24) = 1532, *p* < 0.001; and treatment × time interaction, F(5, 24) = 130, *p* < 0.001. In Caco-2 cells, treatment and time contributed almost equally to total variation, accounting for 41.7% and 40.5%, respectively. This indicates that LDH release in Caco-2 cells was strongly influenced not only by treatment type, but also by exposure duration.

At 24 h, LDH levels in Caco-2 cells were substantially higher than those observed in BJ cells. Control cells showed 2.835 ± 0.134 ng/mL, while treatment groups clustered at markedly elevated values, between 5.017 and 5.875 ng/mL. Thus, most treatments produced a strong early LDH increase in Caco-2 cells, with Mel and MNPs-Mel-MH showing the highest values. By 72 h, LDH levels decreased in several Caco-2 groups. MNPs-MH recorded the lowest value among the Caco-2 treatment groups, reaching 1.115 ± 0.097 ng/mL. In contrast, melittin-containing groups remained elevated, with the MNPs-Mel-MH remaining markedly high at 5.847 ± 0.270 ng/mL. Across all Caco-2 groups, the mean LDH level decreased from 5.07 ng/mL at 24 h to 2.89 ng/mL at 72 h, with a mean difference of 2.18 ng/mL and a 95% CI of 2.07 to 2.30 ng/mL. This suggests that the LDH response in Caco-2 cells was strongest at 24 h, but persisted at 72 h mainly in the melittin-containing conditions. Overall, LDH analysis indicates that Caco-2 cells were more susceptible to early treatment-associated membrane damage than BJ cells, particularly at 24 h. In BJ cells, LDH release was lower and increased more gradually, with the highest 72 h response observed after MNPs-Mel-MH treatment. In Caco-2 cells, most treatments induced high LDH release at 24 h, while at 72 h the response remained especially elevated in the melittin-containing groups, particularly MNPs-Mel-MH. These findings suggest that the combination of melittin functionalization and hyperthermia produced the most sustained cytotoxic effect in Caco-2 cells, while BJ cells displayed a comparatively more limited LDH response.

Previous reports also showed increased LDH levels as an effect of Mel treatment in 3D colorectal cancer spheroids [58], colorectal and gastric cancer cells [59], and ovarian cancer cells [54]. Watanabe et al. noted plasma membrane damage and LDH release in A549 cells when testing Fe_3_O_4_ MNPs at high concentrations [60]. This effect of LDH leakage also depended on the Fe_3_O_4_ nanoparticle size, influencing the toxicity in cell hepatoma [61]. Si et al. tested SiO_2_–alginate–Mel nanoconjugates in an ovarian cancer model and also found high levels of LDH [62]. Other melittin-loaded nanocarriers responsible for such an outcome, consecutive to cell membrane cytotoxicity, were reviewed in [63].

The changes produced by Mel in the groups of cells tested here are explained by the particular properties of this water-soluble, amphiphilic molecule. Many different cellular effects were attributed to Mel, resulting from mechanisms that are not always well explained. In the context of the present study, the well-known non-selective membranolytic mechanism is of particular interest. The literature data showed that in solution, Mel is unstructured, but quickly turns into an α-helix when attached to the membrane–water interface. Melittin molecules bind to negatively charged plasma membrane surfaces, thus deranging phospholipid bilayers [17]. Depending on the concentration, membrane-bound Mel can locate in either a parallel orientation at low concentrations—lying on the membrane surface and interacting with the lipid polar groups—or a perpendicular orientation at high concentrations, when Mel is inserted in the hydrophobic core of the membrane, leading to the formation of pore-like structures [64,65]. Such transient pores, allowing transmembrane conduction of atomic ions (not glucose or larger molecules), are formed at nanomolar concentrations, whereas at the micromolar range, Mel induces stable membrane pore formation, leaking molecules up to 10 kDa. In even higher concentrations, it acts as a detergent, causing the membranes to deteriorate [20] or even to turn into micelles [66]. All of these phenomena ultimately lead to cell lysis. But, in certain conditions, Mel is also able to arrive inside the cells and interact with the intracellular membranes, thus generating multiple and diverse cellular effects. The cytotoxic effects recorded by us here at cellular and subcellular levels were generated by the ability of Mel to permeate (or disintegrate in limited regions) the phospholipid bilayer of the plasma membrane. It arrives at the endoplasmic reticulum as well as the mitochondria, where it interacts with one or both membranes, acting in the same manner as on the plasma membrane. Also, the disturbance of mitochondrial cristae could be explained by Mel’s ability to induce membrane fusions [67], a process during which Mel also aggregates membrane proteins [68]. Another cytotoxic mechanism, secondary to Mel interaction with plasma and mitochondrial membranes, is achieved via mitochondrial signaling pathways, leading to caspase 3-induced apoptosis consecutive to its activation by cytochrome C, which is released into cytosol from the intermembrane space through the Mel pores in the outer mitochondrial membrane [69]. We demonstrate here the increase in caspase 9 levels, which is an activator of caspase 3 in apoptotic cells. Due to its many properties, Mel has been tested with promising results in therapy. Thus, Mel showed anti-inflammatory effects, reviewed by Carpena et al. [70], by blocking toll-like receptors 2 and 4, the cluster of differentiation 14, and the platelet-derived growth factor receptor-β, or by inhibiting interleukins 6 and 8, tumor necrosis factor-α, and interferon-γ [71,72], as well as inducible NO synthase nuclear transcription factor κB and I kappa B, which is a potential new anti-osteoarthritis drug [73]. It was recognized as a potent molecule against various types of cancers [17,70,74,75,76,77,78,79], by interfering with signal transduction and with different regulatory pathways, by stimulating apoptosis or blocking proliferation and angiogenesis, or by increasing tumor cell sensitivity for other treatments, as well as by its direct effect on cell membranes (inducing necrosis). There are too many original articles and reviews published on this topic to be mentioned here, and this is not the subject of our paper.

### 2.6. Apoptosis Induction

**Figure 5:**
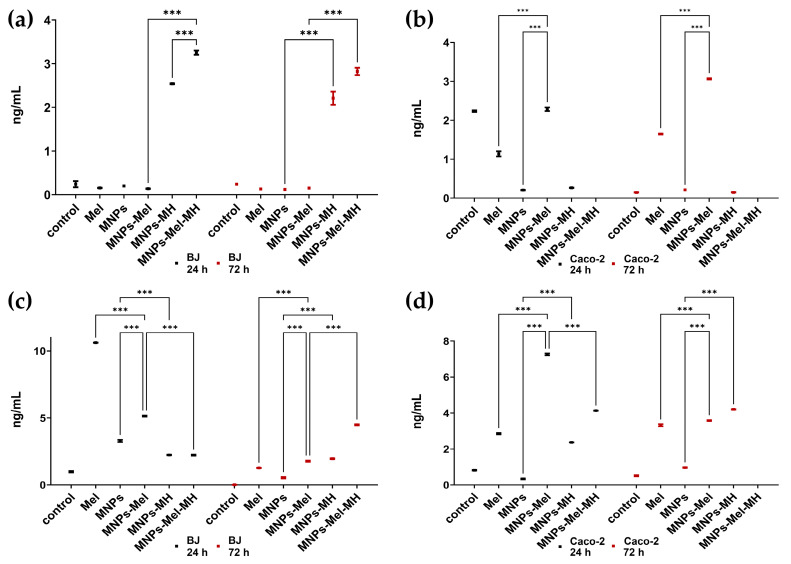
ELISA measurements of caspases 8 (**a**,**b**) and 9 (**c**,**d**) of BJ cells (**a**,**c**) and Caco-2 cells (**b**,**d**) incubated for 24 h with 2.5 µg/mL melittin, and with 50 µg/mL MNPs or MNPs-Mel, with or without exposure to 30 MH at 25 kA/m at 24 h and 72 h. Data (n = 3) are presented as concentration values (mean ± SD). *** = *p* < 0.0001.

Caspase 8 is involved in the triggering of the extrinsic membrane apoptotic pathway, while the activation of caspase 9 is an early event in the intrinsic mitochondrial-dependent apoptotic pathway. In both cell lines tested here, the levels of caspase 9 were strongly increased by the different exposures when compared to caspase 8. This supports the mitochondrial pathway of apoptosis as the most important mechanism for cell death induction in our experimental setting, as previously discussed. Figure 5 shows the levels of caspases 8 (Figure 5a,b) and 9 (Figure 5c,d) of BJ cells (Figure 5a,c) and Caco-2 cells (Figure 5b,d) measured in different experimental conditions.

A global three-way ANOVA was first applied to evaluate the combined influence of treatment, cell type, and time. The analysis showed that caspase 9 levels were significantly affected by treatment, F(5, 48) = 32,361, *p* < 0.0001; cell type, F(1, 48) = 4272, *p* < 0.0001; and time, F(1, 48) = 6319, *p* < 0.0001. The interaction terms were also highly significant, including treatment × cell type, F(5, 48) = 13,118, *p* < 0.0001; treatment × time, F(5, 48) = 22,079, *p* < 0.0001; cell type × time, F(1, 48) = 36,270, *p* < 0.0001; and treatment × cell type × time, F(5, 48) = 6897, *p* < 0.0001. These results indicate that caspase 9 activation was strongly context-dependent, varying according to both the cellular model and exposure duration. In BJ cells, the caspase 9 response was characterized by a pronounced early increase, especially following Mel exposure. Two-way ANOVA confirmed significant effects of treatment, time, and treatment × time interaction, all with *p* < 0.0001. The ANOVA table showed a significant treatment effect, F(5, 24) = 11,057, *p* < 0.0001; a significant time effect, F(1, 24) = 28,009, *p* < 0.0001; and a significant treatment × time interaction, F(5, 24) = 12,501, *p* < 0.0001. The interaction accounted for the largest proportion of variation in the BJ cell analysis, indicating that the treatment effect changed substantially between 24 h and 72 h. At 24 h, the BJ control cells showed a caspase 9 level of 0.980 ± 0.052 ng/mL. MNPs increased caspase 9 to 3.281 ± 0.083 ng/mL, while MNPs-Mel produced a higher value of 5.134 ± 0.043 ng/mL.

The most prominent increase at this time point was observed after Mel treatment, which reached 10.615 ± 0.010 ng/mL. In comparison, the hyperthermia-associated groups showed more moderate values, with MNPs-MH at 2.231 ± 0.026 ng/mL and MNPs-Mel-MH at 2.221 ± 0.044 ng/mL. By 72 h, the pattern in BJ cells shifted. Control values were very low, at 0.011 ± 0.009 ng/mL, while MNPs reached 0.535 ± 0.055 ng/mL. MNPs-Mel and Mel showed values of 1.769 ± 0.051 ng/mL and 1.266 ± 0.015 ng/mL, respectively. The strongest 72 h response was no longer observed with Mel alone, but with MNPs-Mel-MH, which increased to 4.481 ± 0.037 ng/mL. MNPs-MH also remained elevated compared with the control, reaching 1.955 ± 0.030 ng/mL. Overall, mean caspase 9 levels in BJ cells decreased from 4.077 ng/mL at 24 h to 1.669 ng/mL at 72 h, with a mean difference of 2.407 ng/mL and a 95% CI of 2.378 to 2.437 ng/mL.

Caco-2 cells displayed a different temporal profile. While BJ cells showed the strongest overall response at 24 h, Caco-2 cells showed a more pronounced increase at 72 h. Two-way ANOVA confirmed significant effects of treatment, F(5, 24) = 44,472, *p* < 0.001; time, F(1, 24) = 8803, *p* < 0.001; and treatment × time interaction, F(5, 24) = 18,184, *p* < 0.001. Treatment was the dominant source of variation in Caco-2 cells, accounting for 69.0% of total variation, while the interaction accounted for 28.2%, indicating that treatment effects varied considerably between 24 h and 72 h. At 24 h, the Caco-2 control cells showed 0.816 ± 0.021 ng/mL, and MNPs alone remained low at 0.339 ± 0.027 ng/mL. In contrast, MNPs-Mel produced a marked increase in caspase 9, reaching 7.260 ± 0.051 ng/mL.

Mel alone reached 2.856 ± 0.040 ng/mL, while MNPs-MH and MNPs-Mel-MH showed 2.365 ± 0.010 ng/mL and 4.132 ± 0.011 ng/mL, respectively. At 72 h, Caco-2 caspase 9 levels increased further in several treatment groups. MNPs-Mel-MH produced the highest value of the entire caspase 9 dataset, reaching 11.123 ± 0.023 ng/mL (increased with 2164%; data not shown in Figure 5 due to an exaggerated distortion of the graph). MNPs-MH also increased to 4.202 ± 0.012 ng/mL, while MNPs-Mel and Mel reached 3.579 ± 0.028 ng/mL and 3.319 ± 0.055 ng/mL, respectively. The control remained low at 0.514 ± 0.039 ng/mL, while MNPs alone reached 0.962 ± 0.021 ng/mL. Across all Caco-2 groups, the mean value increased from 2.960 ng/mL at 24 h to 3.950 ng/mL at 72 h, with a mean difference of −0.990 ng/mL and a 95% CI of −1.010 to −0.968 ng/mL.

The comparison between BJ and Caco-2 cells highlights a distinct cell-type-specific apoptotic response. At 24 h, BJ cells responded strongly to Mel alone, with caspase 9 reaching 10.615 ± 0.010 ng/mL, compared with 2.856 ± 0.040 ng/mL in Caco-2 cells. BJ cells also showed a higher response to MNPs alone than Caco-2 cells, with 3.281 ± 0.083 ng/mL versus 0.339 ± 0.027 ng/mL. However, Caco-2 cells showed higher caspase 9 levels after MNPs-Mel, 7.260 ± 0.051 ng/mL versus 5.134 ± 0.043 ng/mL, and after MNPs-Mel-MH, 4.132 ± 0.011 ng/mL versus 2.221 ± 0.044 ng/mL. At 72 h, the difference between the two cellular models became more evident. Caco-2 cells showed higher caspase 9 levels than BJ cells in nearly all treatment groups, especially after MNPs-Mel-MH, where Caco-2 cells reached 11.123 ± 0.023 ng/mL, compared with 4.481 ± 0.037 ng/mL in BJ cells. Similar differences were observed for MNPs-MH, 4.202 ± 0.012 ng/mL in Caco-2 versus 1.955 ± 0.030 ng/mL in BJ, and for MNPs-Mel, 3.579 ± 0.028 ng/mL in Caco-2 versus 1.769 ± 0.051 ng/mL in BJ. These results are consistent with the significant treatment × cell type and treatment × cell type × time interactions identified by three-way ANOVA.

Taken together, the caspase 9 data indicate that intrinsic apoptosis-related signaling was strongly influenced by treatment composition and cell type. In BJ cells, Mel alone induced the strongest early caspase 9 response at 24 h, whereas MNPs-Mel-MH became the dominant stimulus at 72 h. In contrast, Caco-2 cells showed a stronger delayed response, with MNPs-Mel-MH producing the highest caspase 9 levels at 72 h. This suggests that Mel-functionalized MNPs combined with MH may preferentially enhance intrinsic apoptotic activation in Caco-2 cells after prolonged exposure, while BJ cells display a stronger early response to free melittin.

Caspase 8 was assessed as a key initiator caspase of the extrinsic apoptotic pathway, which is commonly activated following death receptor signaling and can contribute to downstream executioner caspase activation and programmed cell death. Therefore, caspase 8 measurement was used to evaluate whether Mel, MNPs, MNPs-Mel, and their hyperthermia-associated variants induced apoptosis-related responses in BJ and Caco-2 cells. Caspase 8 levels were significantly affected by treatment, time, and cell type. Three-way ANOVA showed significant main effects of treatment, F(5, 48) = 10,486, *p* < 0.001; cell type, F(1, 48) = 4632, *p* < 0.001; and time, F(1, 48) = 532, *p* < 0.001. Significant interaction effects were also observed for treatment × cell type, F(5, 48) = 4181, *p* < 0.001; treatment × time, F(5, 48) = 506, *p* < 0.001; cell type × time, F(1, 48) = 85.1, *p* < 0.001; and treatment × cell type × time, F(5, 48) = 437, *p* < 0.001. These results indicate that caspase 8 modulation was not uniform across experimental conditions, but depended strongly on the combination of treatment type, exposure duration, and cellular model.

In BJ cells, treatment had a significant effect on caspase 8 levels, F(5, 24) = 3375, *p* < 0.001, as did time, F(1, 24) = 57.75, *p* < 0.001, and the treatment × time interaction, F(5, 24) = 17.31, *p* < 0.001. At 24 h, caspase 8 remained low in the control, MNPs, MNPs-Mel, and Mel groups, ranging from 0.135 ± 0.001 ng/mL in the MNPs-Mel group to 0.238 ± 0.072 ng/mL in the control group. In contrast, a marked increase was observed in the hyperthermia-associated groups, with MNPs-MH reaching 2.539 ± 0.007 ng/mL and MNPs-Mel-MH reaching 3.253 ± 0.044 ng/mL. At 72 h, the same general pattern was maintained. MNPs-MH and MNPs-Mel-MH remained elevated, with values of 2.207 ± 0.154 ng/mL and 2.824 ± 0.086 ng/mL, respectively, while the control, MNPs, MNPs-Mel, and Mel groups remained comparatively low. These data suggest that, in BJ cells, caspase 8 activation was mainly associated with the MH conditions, particularly when MH was combined with melittin-functionalized nanoparticles.

During cell incubation with MNPs-Mel, some of the particles remained in contact for a prolonged time, with the plasma membrane and the attached Mel interacting with the lipid bilayer, generating changes that triggered the activation of caspase 8. On the other hand, the internalized MNPs-Mel transported Mel molecules inside the cytoplasm, and the further application of AMF contributed to the separation of a certain amount of Mel molecules from MNPs. Regardless of the form, Mel arrived at the mitochondria and destabilized their structure (as revealed by the TEM examination), thus participating in the activation of caspase 9 via cytochrome C releasing from the intermembrane space. These results are similar to other studies in the literature. Bee venom treatments increased caspases 8 and 9 and affected colon cancer cell growth in HCT116 and SW480 cells [80], stimulated caspase 8-triggered apoptosis in pancreatic cancer cells [81], and induced (along with separately tested Mel) caspase-dependent apoptosis in prostate cancer cells [82].

Taken together, all of these changes recorded for the various parameters assessed here (cellular viability, and levels of ATP, LDH, and caspases 8 and 9) could be regarded as a consequence of a differentiated ability of the individual cells to internalize the MNPs-Mel since the cells internalized the MNPs-Mel in different amounts, as noted during TEM observations. Moreover, the performed assays concerned molecules released by the cells during the 24 h period consecutive to the incubations for 24 h or 72 h, with a faster release for the more affected cells (some of them did not survive the Mel presence in the environment) or a slower release for the remaining viable cells.

### 2.7. Electron Microscopy Evaluation

We present here detailed TEM results obtained in the different groups of cells consecutive to the 24 h incubation, while relevant data obtained at 72 h are only briefly mentioned in the Supplementary Materials (Figure SI4).

#### 2.7.1. Control Cells and MNPs-Mel Internalization Control

**Figure 6:**
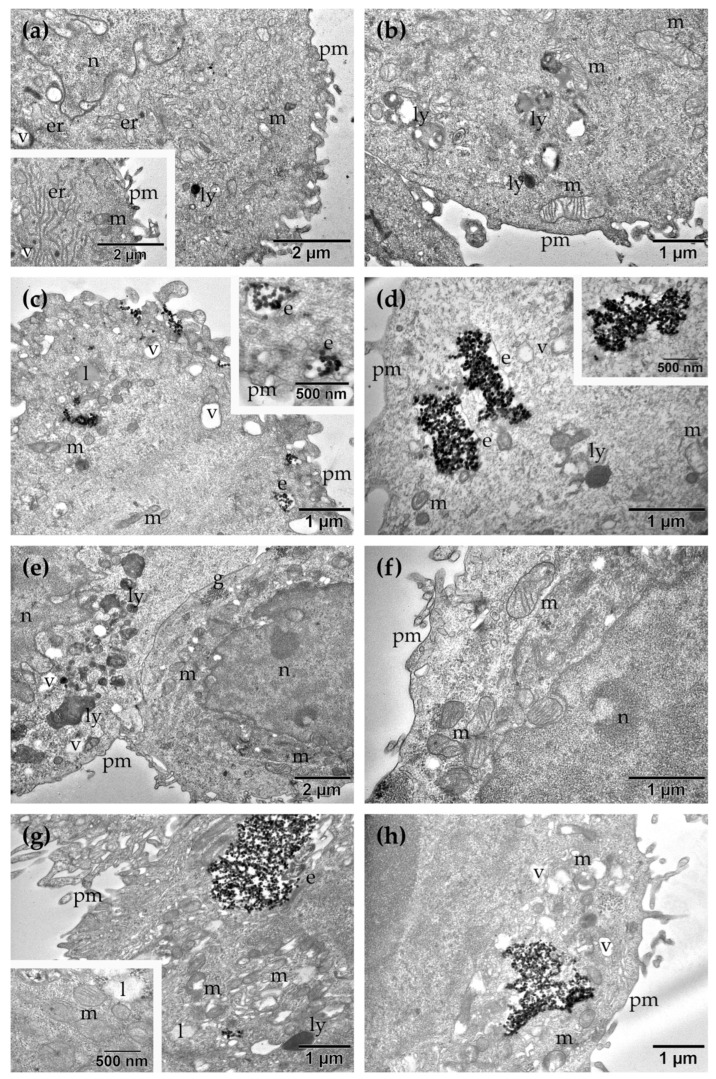
Relevant TEM images of BJ cells: the untreated control (**a**,**b**); cells incubated for 4 h with 50 µg/mL MNPs-Mel for internalization (**c**,**d**); Caco-2 cells the control (**e**,**f**); and cells incubated for 4 h with 50 µg/mL MNPs-Mel for internalization (**g**,**h**) (n = 3). e: endosome; er: endoplasmic reticulum; g: glycogen; l: lipid droplet; ly: lysosome; m: mitochondrion; n: nucleus; pm: plasma membrane; and v: vesicle.

The control BJ cells displayed euchromatic, polymorphic nuclei surrounded by abundant cytoplasm (Figure 6a). Numerous oval mitochondria, with prominent cristae, were noted, as well as several primary and secondary lysosomes, small vesicles (Figure 6a,b), and endoplasmic reticulum profiles grouped in certain areas (Figure 6a). Glycogen granules were uniformly distributed within the cytoplasm (Figure 6a,b). In some cells, lipid droplets or occasional small autophagosomes were present. On the surface, the plasma membrane had numerous thin prolongations (Figure 6a,b). The 4 h incubation with MNPs-Mel proved their early internalization in moderate to high numbers. The MNPs-Mel were observed inside endosomes (inset of Figure 6c,d). It is considered that this internalization process occurred consecutively to a receptor-independent pinocytosis since the literature data does not mention the ability of cells to produce receptors for Mel. When large amounts of MNPs arrived within the cells with endosomes, they mechanically disrupted the endosomal membranes and dispersed in the cytosol, often being observed grouped as they used to be in the endosomes (Figure 6c,d). The cellular ultrastructure remained mostly unchanged following the internalization (Figure 6c,d). The control Caco-2 cells had large, euchromatic nuclei, with irregular contours. In the cytoplasm, numerous mitochondria were found, with a rounded or oval shape and prominent cristae, as well as primary or secondary lysosomes (Figure 6e,f). Several small vesicles were also noted, and glycogen granules and occasional homogenous lipid droplets were found in some cells. The plasma membrane presented thin prolongations (Figure 6e,f). Following the 4 h incubation with MNPs, these were also internalized in high numbers, being visible both in endosomes and dispersed in the cytoplasm (Figure 6g,h). After the exposure to MNPs, some mitochondria showed regions of electron–lucent matrix (Figure 6g,h).

#### 2.7.2. Cells Incubated with Melittin for 24 H

**Figure 7:**
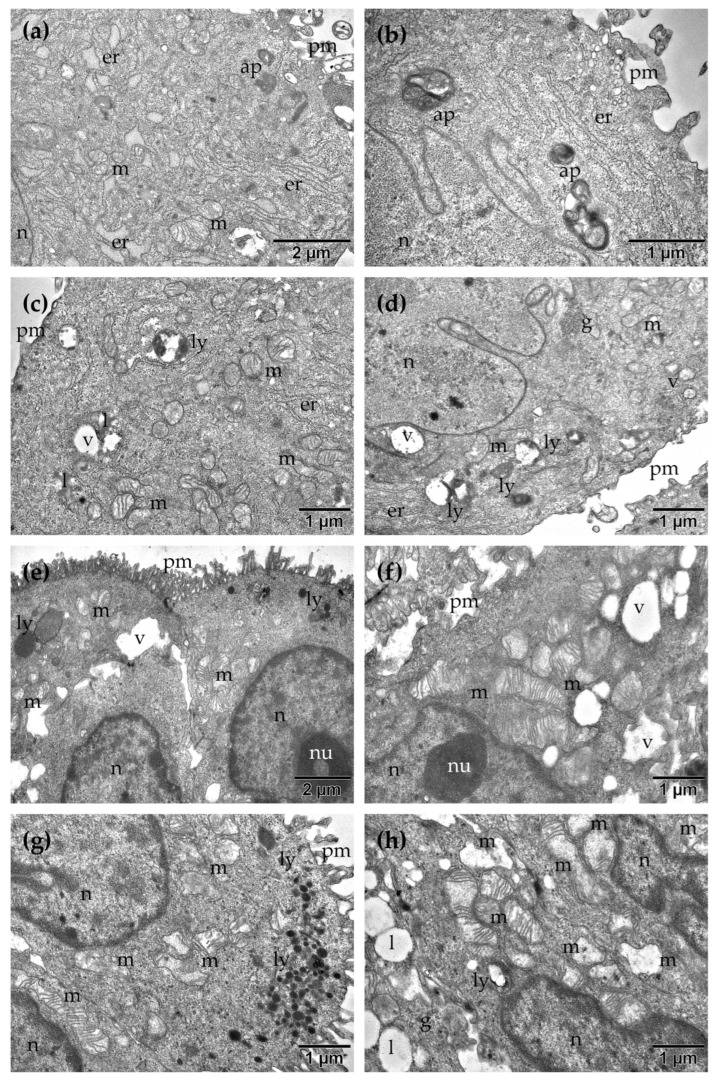
Relevant TEM images of BJ cells (**a**–**d**) and Caco-2 cells (**e**–**h**) incubated for 24 h with 2.5 µg/mL melittin (n = 3). ap: autophagosome; er: endoplasmic reticulum; g: glycogen; l: lipid droplet; ly: lysosome; m: mitochondrion; n: nucleus; nu: nucleolus; pm: plasma membrane; and v: vesicle.

The cells in the BJ-Mel group presented expanded endoplasmic reticulum profiles alongside numerous autophagosomes (Figure 7a,b). A few mitochondria were polymorphic (Figure 7c,d), and many lysosomes had electron–lucent central regions, maintaining their density only in the periphery (Figure 7c,d). Additionally, most lipid droplets observed were heterogeneous, with dense centers (Figure 7c). In rare cells from the Caco-2-Mel group, mitochondria preserved their normal shape but displayed a rarefied matrix (Figure 7e,f). In most cases, mitochondria were deeply affected, with extremely polymorphous shapes and irregular contours, and an electron–lucent matrix. Additionally, some had fewer cristae, while others were completely devoid of cristae (Figure 7g,h). Numerous large vesicles were also present in the cytoplasm (Figure 7e,f,h). Many vesicles resulted from mitochondrial damage, while others could be lipid droplets with very rarified content.

The presence of Mel in low concentration in the culture media surrounding the cells during 24 h of incubation resulted in ultrastructural changes in reduced amplitude in both types of cells, as expected and discussed in Section 2.5. However, the cellular responses were different. They concerned the endoplasmic reticulum and lysosomes in BJ cells, while in Caco-2 cells, the mitochondria were predominantly affected in the context of the lower amount of endoplasmic reticulum generally observed in these cells.

#### 2.7.3. BJ Cells Incubated with MNPs for 24 H and MH Exposure

**Figure 8:**
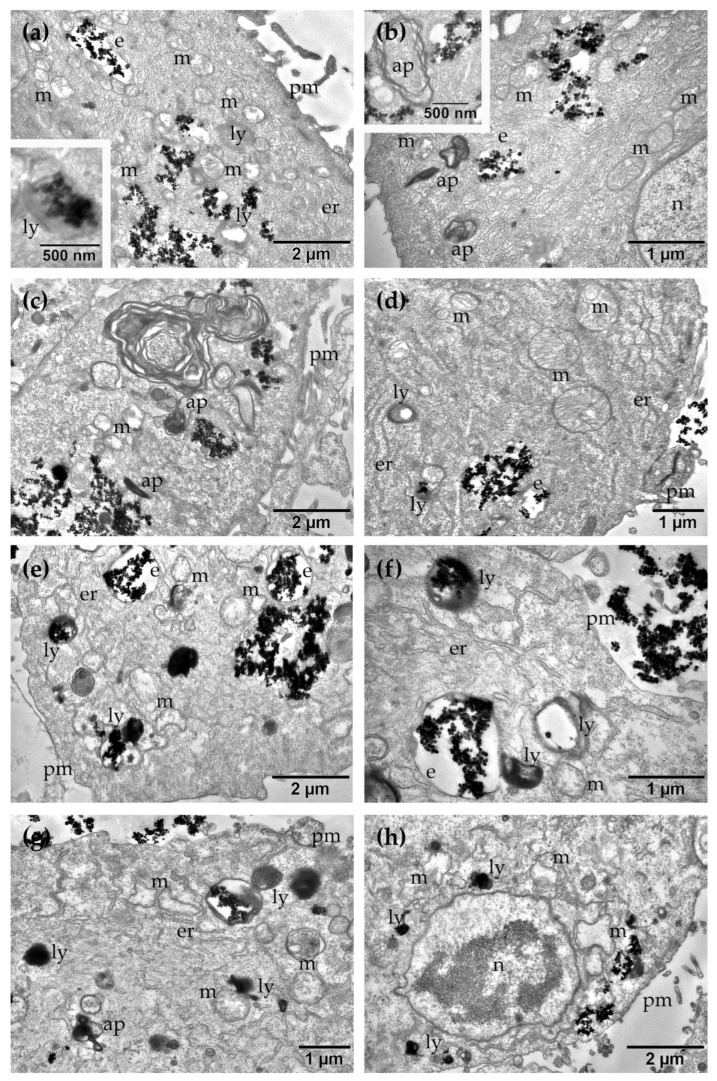
Relevant TEM images of BJ cells incubated for 24 h with 50 µg/mL MNPs in the absence (**a**–**d**) and presence of MH for 30 min at 25 kA/m assessed at 24 h post-MH exposure (**e**–**h**) (n = 3). ap: autophagosome; e: endosome; er: endoplasmic reticulum; ly: lysosome; m: mitochondrion; n: nucleus; and pm: plasma membrane.

The internalization of the MNPs by the BJ cells continued during the exposure for 24 h, and the internalized MNPs were more abundant. Thus, MNPs were still found inside the intact remaining endosomes (Figure 8a,b,d), proving that MH exposure was moderate. Also, the MNPs were visible inside lysosomes as well (inset in Figure 8a), and dispersed freely in the cytoplasm—most of them (Figure 8a,b,d). The presence of MNPs in lysosomes is regarded by us as a consequence of a physiological fusion of MNP-carrying endosomes with lysosomes. The lysosomes loaded with MNPs were, in general, of small sizes, indicating that they accumulated the MNPs by fusion with small endosomes containing a reduced number of MNPs. It remains possible that other, larger lysosomes could have accumulated more MNPs, but they were quickly mechanically destroyed by MNPs with release of the MNPs within the cytosol (which possibly occurred in the case of large endosomes that were destroyed prior to their fusion with lysosomes). Our interpretation is sustained by observation of lysosomes with electron–lucent centers (Figure 8a,d). This is the first report of such a particular ultrastructure of lysosomes consequent to metallic nanoparticle internalization. However, similar lysosomal alterations were observed in DU145 cells following their treatment with ascorbate, menadione, or their combination [83]. While most mitochondria preserved their normal ultrastructure, others had areas of rarefied matrices of various extents or were even completely devoid of cristae (Figure 8a–d). Numerous large autophagosomes were found in some cells (Figure 8b,c). Overall, incubation with MNPs at the used dose had cellular effects of very low amplitude, which is understandable since iron is a biologically relevant element.

This feature has contributed to the perception that iron oxide nanomaterials may offer a more favorable translational profile than many other inorganic nanoparticles, particularly when appropriately formulated. At the same time, the literature makes clear that this advantage is conditional rather than absolute because the biological effects of iron oxide nanoparticles depend strongly on size, dose, coating, aggregation behavior, degradation kinetics, and the characteristics of the exposure environment.

Their safety profile must therefore be interpreted in relation to the complete formulation rather than to the core composition alone [31,42,84,85,86].

BJ cells exposed to MH still contained internalized MNPs inside endosomes (Figure 8e,f) or lysosomes (Figure 8e–g), or free in the cytoplasm (Figure 8e,g,h). However, since the cells were maintained in fresh culture media post-MH exposure (without MNPs), the presence of MNPs in a relatively large number outside the cells indicates their release from cells either by sustained physiological exocytosis or consecutive to plasma membrane damage produced under the influence of MH. On the TEM images, it is not possible to assess how many MNPs are internalized due to the random incidence of the sections. Moreover, unfortunately, when the study was performed, we could not measure the exact amount of intracellular MNPs to precisely describe the dynamics of MNPs in cells. Numerous mitochondria with an electron–lucent matrix and mostly devoid of cristae were noted, as well as several polymorphic ones (Figure 8e–h). A particular aspect was represented by the presence of mitochondria with inner concentric membranes (Figure 8g). In some areas of the cells, the endoplasmic reticulum was slightly expanded (Figure 8e–g), and rare autophagosomes were present (Figure 8g). Additionally, a few lysosomes had electron–lucent central regions (Figure 8e–g).

#### 2.7.4. Caco-2 Cells Incubated with MNPs for 24 H and MH Exposure

**Figure 9:**
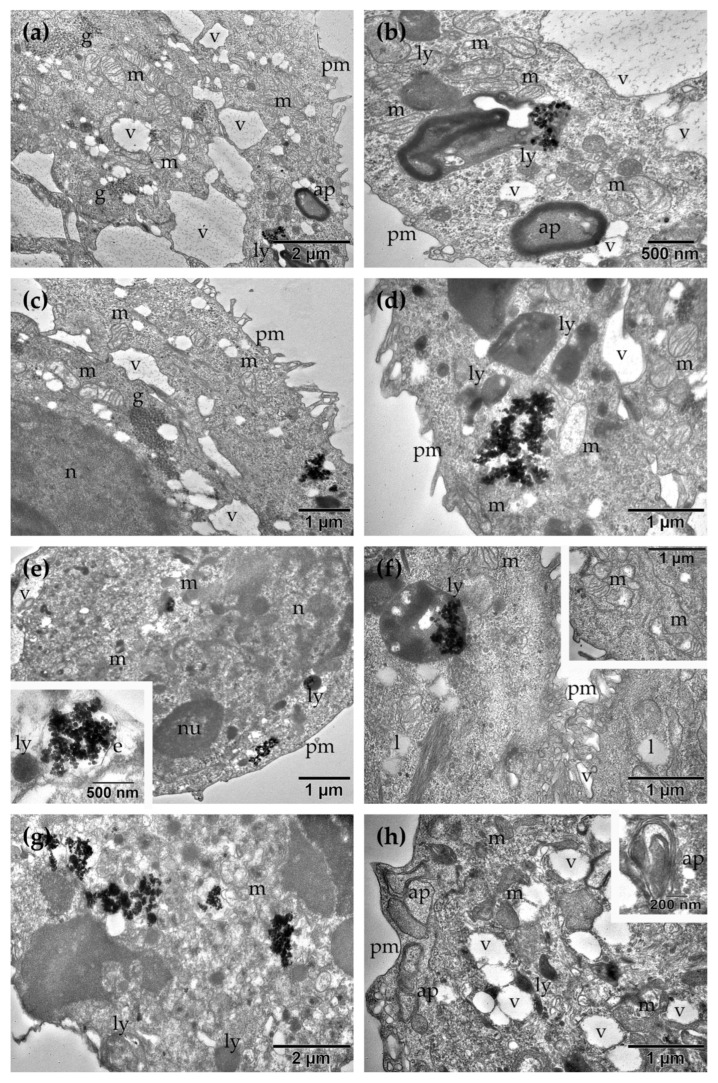
Relevant TEM images of Caco-2 cells incubated for 24 h with 50 µg/mL MNPs in the absence (**a**–**d**) and presence of MH for 30 min at 25 kA/m assessed at 24 h post-MH exposure (**e**–**h**) (n = 3). ap: autophagosome; e: endosome; g: glycogen; l: lipid droplet; ly: lysosome; m: mitochondrion; n: nucleus; nu: nucleolus; pm: plasma membrane; and v: vesicle.

The cells of the Caco-2-MNPs group were highly vacuolated (Figure 9a–d). They contained MNPs inside large secondary lysosomes (Figure 9a,b) or freely dispersed in the cytoplasm (Figure 9c,d). Some mitochondria were long (Figure 9a) with fewer (Figure 9b,c) or no visible cristae (Figure 9d). Occasional autophagosomes were noted (Figure 9a,b). The Caco-2 cells exposed to MNPs and MH also presented MNPs inside rare still-intact endosomes (inset of Figure 9e) in lysosomes with electron–lucent areas (Figure 9f) or free in the cytoplasm (Figure 9e,g). A much lower number of MNPs (compared to the BJ-MNPs-MH group) were noted outside the cells, suggesting that the Caco-2 cells lost their ability to eliminate them, and only a limited number of cells were destroyed by the MH exposure. In these cells, some mitochondria were polymorphic, with fewer or no cristae, with an electron–lucent or condensed matrix (Figure 9f–h). Several autophagosomes of large sizes were noted (Figure 9h), and many cells contained numerous vesicles of various sizes (Figure 9h).

#### 2.7.5. BJ Cells Incubated with MNPs-Mel for 24 H and MH Exposure

In therapeutic systems, Fe_3_O_4_ nanoparticles have been used as carriers for drugs, proteins, peptides, and nucleic acids, while their magnetic properties enable additional possibilities such as magnetically guided retention and magnetic hyperthermia. Thus, Fe_3_O_4_ nanoparticles are not limited to a single role; instead, they can serve as multifunctional material platforms within one nanosystem [20,31,32,42,87]. This multifunctionality is particularly relevant in oncology, where therapeutic limitations often arise from poor localization, low delivery efficiency, and off-target toxicity. In Fe_3_O_4_-based systems, a therapeutic compound may be bound to the nanoparticle surface, entrapped within a coating, or combined with targeting ligands, while the magnetic core remains available for imaging, magnetic retention, or future hyperthermia-based intervention. This capacity to integrate several useful functions within one structure places Fe_3_O_4_ nanoparticles within the broader concept of theranostics [20,32,38,41,88]. A wide variety of coatings have been described for Fe_3_O_4_ nanoparticles, including polyethylene glycol, dextran, chitosan, silica, citrate, phosphonate ligands, lipids, proteins, and synthetic polymers. Because the coating defines the outermost interface between the nanoparticle and the biological environment, it strongly influences cellular interaction, circulation, persistence, and therapeutic behavior [37,43,89,90,91].

**Figure 10:**
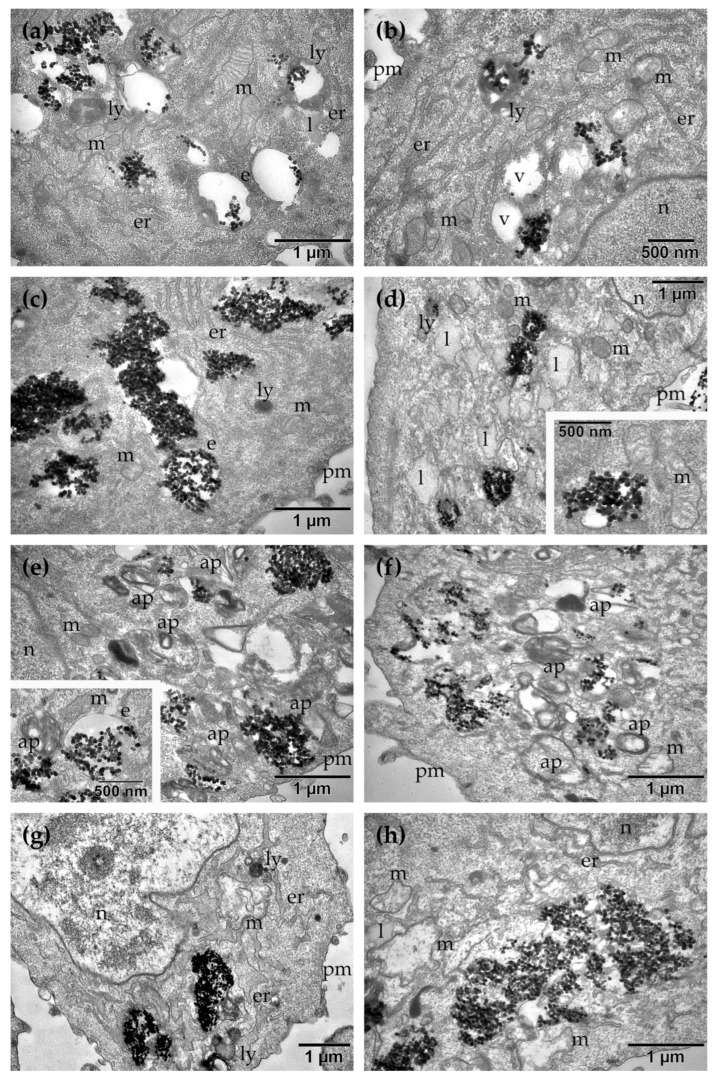
Relevant TEM images of BJ cells incubated for 24 h with 50 µg/mL MNPs-Mel in the absence (**a**–**d**) and presence of MH for 30 min at 25 kA/m assessed at 24 h post-MH exposure (**e**–**h**) (n = 3). ap: autophagosome; e: endosome; er: endoplasmic reticulum; l: lipid droplet; ly: lysosome; m: mitochondrion; n: nucleus; pm: plasma membrane; and v: vesicle.

In our study, the MNPs-Mel were responsible for important cellular changes in the tested cells, which is important to understand the results reported above. The BJ cells in this group presented abundant internalized MNPs-Mel, which were visible and mainly free in the cytosol, either grouped or dispersed (Figure 10a–d). The presence of MNPs-Mel was also noted inside endosomes, in very low (Figure 10a) or high numbers, filling the endosome (Figure 10c). Figure 5c also shows a large MNPs-Mel containing endosomes with a disrupted membrane, releasing its content into the cytosol. The MNPs-Mel were also included in lysosomes (Figure 10a,b,d), with different degrees of degeneration and electron–lucent centers (Figure 10a,b). In general, mitochondria had normal aspects, but also rare polymorphic mitochondria or mitochondria with no visible cristae were identified (Figure 10a,b,d). The inset in Figure 5d shows such a mitochondrion with a tendency to become polymorphous in the immediate proximity of a group of MNPs-Mel. The last ultrastructural change in this group was represented by the irregular shapes of lipid droplets (Figure 10d). MNPs-Mel released from endosomes interacted with various organelles, with mitochondria being mainly responsible for the reported effects. On the other hand, a certain amount of Mel could have arrived in the cells by crossing the perforated plasma membrane under the action of the surrounding MNPs-Mel. Regardless of the mechanism, only a limited amount of Mel acted intracellularly and produced limited effects in the examined cells. However, it remains possible that other cells were more severely damaged, thus explaining the results reported for the cell viability tests. BJ cells of this group exposed to MH still contained internalized MNPs-Mel in large amounts, inside rare endosomes or freely dispersed in the cytoplasm (Figure 10e–h), sometimes being released from disrupted endosomes (Figure 10f). A few mitochondria had normal ultrastructure (Figure 10e) or contained a reduced number of cristae on the background of an electron–lucent matrix (Figure 10f); most mitochondria showed aberrant shapes, with an accentuated irregular contour, electron–lucent matrix, and few or no cristae (Figure 10g,h). Expanded endoplasmic reticulum profiles were noted (Figure 10g), and very numerous autophagosomes of different sizes were present in the cells (Figure 10e,f).

The MH exposure released more MNPs-Mel from the endosomes, as well as more Mel from MNPs, inside and outside the endosomes. Therefore, the ultrastructural alterations were more severe in the cells of this group than in the cells not exposed to MH. The most affected organelles were mitochondria, thus confirming the lower capacity of these cells to produce ATP (see Section 2.4). On the other hand, the reduced amount of ATP resulted in our study from the lower number of cells surviving the experimental treatments, as shown by the viability tests. A similar ultrastructural aspect of the mitochondria (swelling, accompanied by a decrease in the number of cristae) was noted in HeLa cells treated with doxycycline-loaded carbonated hydroxyapatite nanoparticles [92]. Additionally, silver nanoparticles in concentrations of 6 μg/mL were proven cytotoxic in HepG2 cells, leading to swollen mitochondria and the disorganization of their inner membranes [93]. The ability of Mel to alter in a short time, and depending on the dose, the mitochondrial ultrastructure and the shape and number of cristae, was previously reported in another in vitro study where the adrenocortical mitochondria (with well-organized, parallel tubular cristae) were incubated with multiple Mel solutions [94]. Other in vivo results showed relevant ultrastructural changes in the adrenocortical mitochondria triggered by high doses of honeybee venom [95]. The autophagosomes present in high numbers represent additional evidence supporting the alteration of cellular structures by Mel.

#### 2.7.6. Caco-2 Cells Incubated with MNPs-Mel for 24 H and MH Exposure

**Figure 11:**
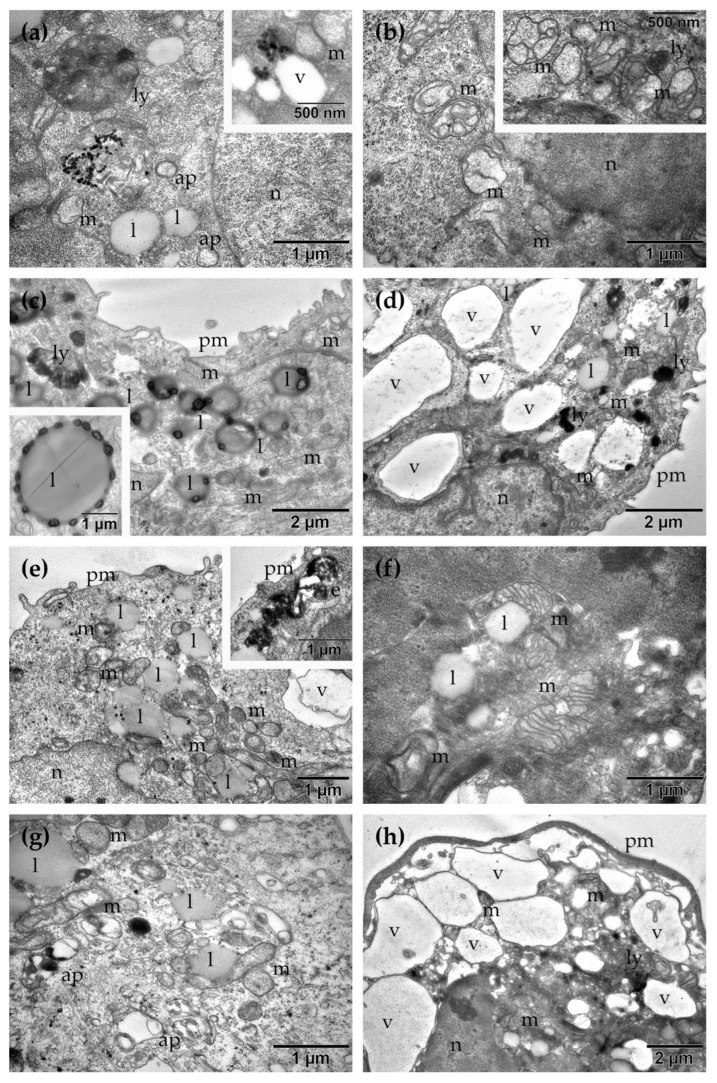
Relevant TEM images of Caco-2 cells incubated for 24 h with 50 µg/mL MNPs-Mel in the absence (**a**–**d**) and presence of MH for 30 min at 25 kA/m assessed at 24 h post-MH exposure (**e**–**h**) (n = 3). ap: autophagosome; e: endosome; l: lipid droplet; ly: lysosome; m: mitochondrion; n: nucleus; pm: plasma membrane; and v: vesicle.

The Caco-2 cells in this group contained only low amounts of internalized MNPs-Mel dispersed within the cytoplasm (Figure 11a). But it is important to note that these cells are still viable ones, with changes less severe and/or delayed compared to those triggering cellular death reported in viability assays. However, all mitochondria were altered. They were rounded or polymorphic, with few and modified cristae, or with no visible cristae (Figure 11a–d). Secondary lysosomes of various sizes were observed in many cells (Figure 11a–c), as well as relatively small autophagosomes (Figure 11a). A particular and interesting ultrastructural detail in this group was the presence of numerous lipid droplets with rounded, electron-dense structures around their rim (Figure 11c), indicating an interference of Mel with lipid metabolism as previously shown in ovarian cancer cells [96]. Also, most cells had numerous large vesicles in their cytoplasm (Figure 11d). The Caco-2 cells exposed to MNPs-Mel and MH contained a reduced number of internalized MNPs-Mel dispersed within the cytoplasm or visible in endosomes—with some of them only partly surrounded by membrane (Figure 11e). The main ultrastructural finding in this group was represented by alteration of all mitochondria: some were long or polymorphic, either with a heterogeneous, granular matrix and devoid of cristae (Figure 11e-g), or with a homogenous matrix and disorganized cristae (Figure 11f,h). Several small autophagosomes were present (Figure 11g). Most cells showed profound cytoplasmic alteration, making it difficult to distinguish and recognize the various organelles (Figure 11g), and some other cells showed extensive vacuolation (Figure 11h).

Transmission electron microscopy was rarely employed in assessing the effects of internalized nanomaterials, and there are no such reports on the Mel-functionalized nanomaterials. Therefore, our paper brings new and interesting complementary data useful to further understanding the mechanisms that triggered the molecular reactions of low doses of Mel, revealed by the other methods. Thus, TEM was used here as the most adequate method to visualize the actual internalization of the MNPs-Mel, the dynamics of the process, as well as the fine ultrastructural changes in the most relevant organelles.

While the single notable effect of MNPs was the generation of numerous or large autophagosomes (evidence supporting a low-level alteration of cellular structures and stimulation of autophagy mainly in the BJ cells), Mel delivered inside the cells by the MNPs deeply altered mitochondria, lysosomes, or endoplasmic reticulum. TEM also enabled us to discriminate the actual effects of the Mel transported intracellularly by the MNPs from those of the Mel acting from outside the cells. Finally, TEM allowed us to correlate and explain the molecular results reported here. Unfortunately, since the analyzed pellets contained only the remaining viable cells, we could not identify the actual ultrastructural reactions triggering cellular death. However, this investigation suggested that the reduced levels of ATP and the high levels of caspase 9 recorded consecutively to the incubation of cells with MNPs-Mel and exposure to MH possibly resulted from a direct effect of Mel on the mitochondrial membranes, as well as from an indirect one, after lysosomal disruption.

Despite the large amount of original data provided, the current study has several limitations. On the one hand, we tested the effects of MNPs-Mel under MH conditions on a single cell line (Caco-2) as a well-established model with extensive use in cancer research. These cells are particularly useful for studying intestinal epithelial biology and colorectal adenocarcinoma-related mechanisms [97], but they may not fully capture the diversity and complexity of CRC observed in vivo. The use of a single CRC cell line limits the generalizability of our findings. Although Caco-2 cells originate from a human colorectal adenocarcinoma [98] and are widely employed as an in vitro CRC model [99], they exhibit extensive spontaneous differentiation toward an enterocyte-like phenotype and form polarized epithelial monolayers [13]. While the model offers reproducibility and well-characterized growth, its differentiated phenotype limits its ability to fully represent colorectal tumor heterogeneity and may differ in proliferation, transport, and metabolism from other models. Thus, the present findings on Caco-2 cells cannot be assumed to apply to all colorectal cancers or other tumor types and should be seen as early in vitro insights rather than a complete representation of CRC biology. Therefore, further studies using additional cancer cell lines and in vivo models are required to determine the generalizability of the observed effects. On the other hand, the BJ fibroblasts were used in the present study as a representative non-transformed human cell line to obtain a preliminary assessment of the effects of MNPs-Mel on normal cells. No additional toxicity evaluations in other healthy cell types or in vivo models were performed. We acknowledge that the use of a single normal cell line does not fully characterize the biocompatibility of the formulation, and future studies should include broader toxicological assessments in multiple normal cell types and animal models to establish the safety profile of MNPs-Mel.

Another important limitation of our study resulted from the too large amount of data collected, forcing us to limit the investigations to the use of a single dose of Mel/MNPs/MNPs-Mel. We are fully aware that a demonstration of a dose-dependent (not only time-dependent) effect would be extremely interesting for the readers.

## 3. Materials and Methods

### 3.1. Synthesis of MNPs

Magnetic nanoparticles (MNPs) were synthesized using the following analytical-grade reagents without further purification: iron(III) acetylacetonate [Fe(acac)_3_] (Merck Schuchardt OHG, Hohenbrunn, Germany), ethylene glycol (EG) (Carl Roth GmbH, Karlsruhe, Germany), oleic acid (OA), and dibenzyl ether (DBE) (Sigma-Aldrich, Steinheim, Germany). The synthesis process began by magnetically stirring a reaction mixture containing 2 mmol of Fe(acac)_3_, 12 mmol of OA, 0.5 mL of EG, and 20 mL of DBE at 50 °C for 30 min (500 rot/min) using a magnetic stirrer (Heidolph Instruments GmbH & Co. KG, Schwabach, Germany). This mixture was then transferred to a glass vessel, which was placed inside a stainless-steel container. To remove oxygen, nitrogen was bubbled through the mixture for 5 min. The container was sealed tightly with a Teflon gasket and secured with five screws. The sealed container was placed in an oven (Nabertherm GmbH, Lilienthal, Germany) equipped with a temperature controller (JUMO dTron 316, JUMO GmbH & Co. KG, Darmstadt, Germany). The reaction mixture was initially heated to 200 °C at a rate of 6 °C/min and maintained at this temperature for 2 h. Subsequently, the temperature was increased to 300 °C at a rate of 10 °C/min and held for an additional hour. The resulting black product was isolated using a neodymium magnet and washed multiple times with a 40 mL mixture of ethanol (Chemical, Iași, Romania) and double-distilled water, employing 15 min ultrasonication, using a water bath sonicator Elmasonic P 70 H (Elma Schmidbauer GmbH, Singen, Germany) operating at 37 kHz, with an effective acoustic power of 100 W in continuous mode, and magnetic separation between washes. Finally, the MNPs were dispersed in 100 mL of an aqueous solution of citric acid (Sigma-Aldrich, Steinheim, Germany) at a concentration of 0.1 M. The dispersion was mechanically stirred (Nahita, Auxilab S.L., Beriain, Spain) on a heating plate (Witeg Labortechnik GmbH, Wertheim, Germany) at 80 °C for 1 h to facilitate the grafting of citrate molecules onto the MNP surfaces [100]. The MNPs were subsequently washed with double-distilled water using three cycles of ultrasonication (15 min each), followed by magnetic separation. They were then redispersed in 20 mL of double-distilled water. To determine the concentration of the MNPs, four 1 mL samples were dried: the MNPs were magnetically separated, and the remaining water was removed using a rotary evaporator (Heidolph Instruments GmbH & Co. KG, Schwabach, Germany) over an 8 h period. The mass of the resulting powder was calculated by subtracting the mass of the empty vials from the mass of the vials containing the powder. This powder was subsequently used for diffraction and magnetic analysis. Finally, the citrate-coated MNPs were dispersed in the required volume of double-distilled water to achieve a concentration of 4 mg/mL and then stored in a glass container.

### 3.2. Characterization of MNPs

The morphology and size of MNPs were evaluated using a JEOL JEM-100CX II (Jeol, Tokyo, Japan) transmission electron microscope, operating at 80 kV, equipped with a MegaView G3 camera (Emsis, Münster, Germany) running with Radius 2.1 software (Emsis). Samples were prepared by placing one drop of water suspension of MNPs (10 µg MNPs/mL) on a carbon-coated copper grid. After 5 min, the excess water was removed with filter paper. The size distribution of MNPs was determined through manual measurement of around 650 MNPs using Image J 1.54f (National Institute of Health, University of Wisconsin, Madison, WI, USA).

The UV-Vis absorption spectra of all samples were recorded with a T92+ UV-VIS Spectrophotometer (PG INSTRUMENTS, Leicestershire, UK) using standard quartz cells at room temperature, over a spectral range between 190 nm and 900 nm and a spectral resolution of 2 nm.

The crystalline structure of NFs was determined by X-ray powder diffraction conducted in a Bruker D8 Advance diffractometer using Cu Kα radiation (Bruker AXS GmbH, Karlsruhe, Germany).

Magnetic measurements at 300 K were carried out in a vibrating sample magnetometer device from Cryogenic Limited (London, UK) under applied magnetic fields from 0 to ±2 T.

The heating efficiency was evaluated using a magnetic hyperthermia system, the Easy Heat 0224 from Ambrell (Scottsville, NY, USA), which was equipped with an optical fiber temperature sensor (0.1 °C accuracy). A volume of 0.5 mL of MNPs was placed in the center of an 8-turn coil using a thermally isolated Teflon holder and then submitted to an AC magnetic field with fixed frequency (355 kHz) and variable amplitude (H) (10–60 kA/m). Details of specific absorption rate (SAR) calculations are provided in the Supplementary Materials (Section SI1).

The experimental data were processed and the plots were generated using OriginPro 2022 Academic software (OriginLab Corporation, Northampton, MA, USA).

### 3.3. MNPs Functionalization with Melittin

An amount of 1 mL of an aqueous Mel solution containing 116.34 (±4.41) μg Mel was combined with 1 mg of MNPs previously coated with citrate. The mixture was ultrasonicated for 10 min and then mechanically shaken for an additional 10 min. Following this, the samples were exposed to a magnetic field to separate the MNPs, and the supernatant was analyzed using UV-Vis spectroscopy. It is important to note that a minimum of 5 min is required to ensure complete separation of the MNPs from the solution. Insufficient separation time may result in residual MNPs contributing to the UV-Vis spectrum in the UV region, potentially interfering with the accurate quantification of Mel (Figure SI2c). The coupling of Mel to the MNPs is hypothesized to occur through electrostatic interactions (adsorption) between the positively charged Mel and the negatively charged citrate-coated MNPs. For cell exposure experiments, a dose of 50 µg/mL of MNPs-Mel was used, which corresponded to 2.5 µg/mL of adsorbed melittin.

### 3.4. Cell Cultures

Cell cultures used for the assay were two human cell lines: dermal fibroblasts (BJ, ATCC CRL-2522™, Gaithersburg, MD, USA) and Caucasian colon adenocarcinoma Caco-2 (ECCAC, Sigma Aldrich, Co., Heidelberg, Germany). The medium used for the cell cultures was Dulbecco’s modified Eagle medium (DMEM), supplemented with 10% fetal calf serum, 50 µg/mL gentamicin, and 5 ng/mL amphotericin, all from Biochrom AG (Berlin, Germany). Cultures were fed twice weekly.

### 3.5. Viability Assay

Cell viability was assessed by colorimetry using the CellTiter 96^®^ AQueous Non-Radioactive Cell Proliferation Assay (Promega Corporation, Madison, WI, USA) according to the manufacturer’s instructions. Readings were obtained using a SpectraMax iD3 Multi-Mode Microplate Reader (Molecular Devices, San Jose, CA, USA) at 540 nm.

BJ and Caco-2 cells were seeded at a density of 10^4^/well in 96-well plaques (TPP, Transadingen, Switzerland) for 24 h, and then exposed for 24 h to Mel (0–20 µg/mL), MNPs, and MNPs-Mel (0–400 µg/mL) dispersed in medium. All solutions were prepared immediately before cell incubation. All experiments were performed in triplicate. Cultures exposed to the medium were used as controls. The results are presented as the % of the untreated control.

According to the viability assays, the dose of free Mel selected for incubation of cells was 2.5 µg/mL, corresponding to the goals of this in vitro study, since it resulted in less than 50% viability in Caco-2 cells at 72 h. This dose aligns with effective in vitro ranges (1–10 µg/mL) [17,22,24] but is 100–1000× lower than the in vivo doses (1–5 mg/kg) [74,75,78]. The concentration of melittin adsorbed on MNPs was 54.57 µg Mel/mg MNPs, as proved by UV-Vis spectroscopy. Thus, 50 µg of MNPs contained about 2.5 µg of Mel, which is equivalent to the concentration of the free Mel tested, and confirmed as effective by the viability assays. Therefore, the dose of MNPs-Mel selected for magnetic hyperthermia experiments was 50 µg/mL. Our dose is 100–400 times lower than clinical intratumoral MH doses (5–20 mg/mL) [30,41,88], with SAR (110–2900 W/g) comparable to approved systems.

### 3.6. In Vitro Magnetic Hyperthermia

An 8-turn coil connected to a commercially available magnetic hyperthermia system, the Easy Heat 0224 from Ambrell (Scottsville, NY, USA), was used, working at a frequency of 355 kHz and variable amplitudes. A preliminary investigation was conducted by testing the amplitudes of 15, 20, 25, and 30 kA/m to determine an optimal intensity of AMF for the combined therapy of Mel intracellular delivery by MNPs-Mel and the MH mediated by the nanoparticles. The viability assessment was performed at 24 h and 72 h to select the most relevant time exposure required for the cell death induction. The aim was to generate a toxic effect of Mel (viability between 50 and 70% of the untreated control) by AMF intensity and exposure time that would also allow us to test the pathway of cellular killing and the assessment of cell ultrastructural alterations. According to the results presented in Figure SI3, the value of 25 kA/m was chosen for the hyperthermia experiments since the result of the product between it and the AMF frequency (H∙f = 8.9 × 10^9^ A·m^−1^·s^−1^) was below the newly accepted biological limit (H∙f = 9.59 × 10^9^ A·m^−1^·s^−1^ [35]).

For the main experiment, cells were settled in culture Petri dishes (Ø = 9 cm) at a density of 4 × 10^4^/cm^2^ for 24 h, exposed to MNPs and or MNPs-Mel (50 µg/mL for 24 h or 72 h), and then washed and collected by trypsinization. The cell pellet (approx. 5 × 10^5^ cells) was resuspended in 400 µL of fresh medium. The cellular suspension was equally divided into two aliquots of 200 µL each. One of the aliquots was kept in a water bath at 37 °C (negative control), while the other aliquot was exposed to an AMF for 30 min. Following AMF exposure, cells were seeded in fresh medium on 96-well plaques or on Petri dishes, depending on the parameter assessed, for an additional 24 h, and then collected and processed according to the previously described protocol.

### 3.7. Experimental Groups

The following experimental groups were taken into consideration for both BJ and Caco-2 cells: the control group (untreated cells), the Mel group (cells incubated for 24–72 h with 2.5 µg/mL melittin), the MNPs group (cells incubated for 24–72 h with 50 µg/mL MNPs), the MNPs-Mel group (cells incubated for 24–72 h with 50 µg/mL MNPs-Mel), the MNPs-MH group (cells incubated for 24–72 h with 50 µg/mL MNPs and exposed to 30 min MH at 25 kA/m), and the MNPs-Mel-MH group (cells incubated for 24–72 h with 50 µg/mL MNPs-Mel and exposed to 30 min MH at 25 kA/m). At the end of the incubation time (with Mel, MNPs, and MNPs-Mel) and consecutive to the 24 h incubation period post-MH exposure, the culture media were collected for the various molecular analyses, the cells were detached by trypsinization, centrifuged for 7 min at 500× *g*, and the cell-containing pellets were processed for TEM.

### 3.8. Toxicity Assays

The control BJ and Caco-2 cells and the cells incubated for 24 h with 2.5 µg/mL melittin, 50 µg/mL MNPs, or MNPs-Mel, with or without exposure to 30 min MH at 25 kA/m, were seeded at a density of 10^3^/well in 96-well plates in fresh medium in normal cell culture conditions for 72 h. ATP levels in the cells, which are used to assess cell viability and mitochondrial activity (n = 4), were measured by using the CellTiter-Glo Luminescent Cell Viability Assay (Promega, Madison, WI, USA), according to the manufacturers’ instructions. Readings were carried out in luminescence using the SpectraMax iD3 Multi-Mode Microplate Reader (Molecular Devices, San Jose, CA, USA). Data are presented as the % of untreated controls (cells exposed to medium, with AMF). To further complete the toxicity evaluation, the level of lactate in the supernatant of the cells (medium) was measured at 24 h and 72 h consecutive to a 24 h incubation with Mel, with MNPs and MNPs-Mel with or without 30 min MH at 25 mA/m, by using the Fine Test K060 colorimetric lactate assay kit (Fine Biotech Co., Wuhan, China), as indicated by the producer. These experiments were carried out in triplicate.

### 3.9. ELISA

The levels of caspase 8, which is mainly involved in the membrane-associated apoptosis pathway, and of caspase 9, triggering the mitochondrial apoptotic pathway, were measured by ELISA from cell medium to determine the apoptosis induction mechanism. Caspase 8 (EH0682) and 9 (EH0650) ELISA Immunoassay kits from Fine Test (Fine Biotech Co., Wuhan, China) were used as indicated by the manufacturer, and data was expressed as ng/mL. Media used for the caspases and LDH assessment were first collected, samples were then washed, and fresh medium was added. Afterwards, the cells were treated with the CellTiter 96^®^ AQueous Non-Radioactive Cell Proliferation Assay (Promega, Madison, WI, USA) for the viability assessment and, respectively, the CellTiter-Glo Luminescent Cell Viability Assay (Promega, Madison, WI, USA) for the ATP measurement. To minimize potential assay variability, all CellTiter-Glo measurements were performed using identical, fresh culture medium compositions and volumes across experimental groups. In addition, appropriate cell-free controls were included to assess potential interference of MNPs-Mel with the luminescent ATP detection system.

### 3.10. Statistical Analysis

Statistical analysis was performed using GraphPad Prism 11.02 (Insight Partner, New York, NY, USA). For the viability and ATP toxicity tests, the analyses were performed separately at each time point. Time was therefore not included as an additional factor in the factorial model. This approach was selected because the primary objective was to evaluate, at each analyzed exposure time, whether nanoparticle functionalization and MH independently or interactively affected cell viability. The analysis included MNPs (50 µg/mL), MNPs-MH (50 µg/mL), MNPs-Mel (50 µg/mL), and MNPs-Mel-MH, which form a complete 2 × 2 factorial design. Therefore, a two-way ANOVA was used to evaluate the main effect of nanoparticle functionalization, the main effect of AMF exposure, and the interaction between functionalization and AMF exposure. Nanoparticle functionalization was defined as the presence or absence of Mel adsorption, while MH exposure was defined as absent or present. This model allowed the main effects of functionalization and MH, as well as their interaction, to be evaluated. The interaction term was considered particularly relevant because it indicates whether the effect of MH on cell viability differs according to whether the MNPs are functionalized with Mel. The untreated control and free Mel groups were not included in the factorial ANOVA because they do not represent combinations of the two nanoparticle-related factors. These groups were retained as reference conditions for descriptive interpretation and for planned comparisons. For the LDH and the caspase 8 and 9 assays, a three-way ANOVA was used because the analysis included three independent factors: cell type, time, and treatment. In contrast to the focused two-factor analysis used for the viability and ATP assays, this model was selected to determine whether treatment-related changes in LDH release and caspase activity varied between BJ fibroblasts and Caco-2 cells and whether these differences depended on the analyzed time point. The model evaluated the individual contribution of each factor, the two-way interactions between factors, and the three-way interaction. The two-way interactions assessed whether the effect of one factor depended on another, while the three-way interaction evaluated whether the treatment-dependent response differed according to both cell type and time.

The assumptions underlying the ANOVA models were assessed prior to interpretation of the results. Given the small number of biological replicates per group (n = 3), normality was evaluated primarily through visual inspection of the data distribution and model residuals rather than relying solely on formal normality tests. Homogeneity of variances was assessed using the diagnostics available in GraphPad Prism. No substantial deviations from normality or equality of variances were observed. Because all experimental groups contained the same number of biological replicates, the balanced design was considered appropriate for ANOVA.

### 3.11. Nanoparticle Internalization and Ultrastructural Evaluation of Cells

At the end of the incubation intervals, and after a 5 min trypsinization, the various groups of BJ and Caco-2 cells were suspended in 1.5% osmium tetroxide (Sigma-Aldrich, St. Louis, MO, USA) solution in 0.15 M phosphate buffer (pH 7.4) for a 1.5 h fixation, and then centrifuged for 7 min at 500× *g*. The samples were next dehydrated in acetone series (50–100%, 30 min each) and infiltrated with acetone solutions of EMBed-812 (Electron Microscopy Sciences, Hatfield, PA, USA) of increasing concentrations (30%, 50%, and 70%; 1 h each and 100% overnight). Sections of 60–70 nm were cut with an Ultra 45◦ diamond knife (DiATOME AG, Nidau, Switzerland) on a Bromma 8800 ULTRATOME III ultramicrotome (LKB Produckter AB, Stockholm, Sweden; Bromma, Sweden). They were collected on 300 mesh copper grids (Agar Scientific Ltd., Stansted, UK) and contrasted for 5 min with 13% ethanol solution of uranyl acetate (Merck, Darmstadt, Germany) in ethanol 50%, and for 5 min with 2.8% aqueous solution of lead citrate (Fluka AG, Buchs, Switzerland). Sections were examined with the same transmission electron microscope mentioned in Section 3.2.

## 4. Conclusions

The results reported here show that Mel functionalization of MNPs increased the biological activity of Mel against Caco-2 cells at the low tested dose of 2.5 µg/mL. The AMF hyperthermia further enhanced this effect, and among the tested groups, the strongest and most consistent reduction in viability, metabolic activity, as well as the highest level of ultrastructural alterations was observed in Caco-2 cells after treatment with MNPs-Mel coupled with MH. The BJ control cells proved to be less sensitive to all the administered experimental treatments. Our work provides a promising proof-of-concept, and the presented findings support the potential of combining Mel-functionalized MNPs with MH as an antitumoral strategy with in vivo applications, benefiting the important advantage of their magnetic guidance toward the tumor.

## Data Availability

The original contributions presented in this study are included in the article/Supplementary Materials. Further inquiries can be directed to the corresponding author.

## Supplementary Materials

The following supporting information can be downloaded at: https://www.mdpi.com/article/10.3390/molecules31122171/s1, Figure SI1: Typical temperature change ΔT versus time curves fitted with the Box–Lucas equation (blue curves) of MNPs dispersed in water at concentration of 0.5 mg/mL, recorded as a function of H (10–60 kA/m) at a frequency of 355 kHz; Figure SI2: (a) UV-Vis absorption spectra of aqueous melittin solutions at varying concentrations (1250 to 15,625 µg/mL). (b) Calibration curve showing the relationship between absorbance at 280 nm and melittin concentration (ranging from 1.250 to 15.625 µg/mL) in water, which was used to determine melittin concentration in subsequent experiments. The red line indicates the linear fit of the experimental data. (c) UV-Vis spectra of aqueous melittin solutions and supernatant solutions collected at different time intervals following the magnetic separation of melittin-coated magnetic nanoparticles from the solution; Figure SI3: Viability of Caco-2 cells incubated with 50 µg/mL MNPs (a) and MNPs-Mel (b) and exposed to different MH conditions; Figure SI4: Relevant TEM images of BJ and Caco-2 cells recorded consecutive to different experimental treatments of 72 h.

## Abbreviations

The following abbreviations are used in this manuscript:

AMF	Alternating magnetic field
CRC	Colorectal cancer
Fe(acac)_3_	Iron(III) acetylacetonate
Mel	Melittin
MH	Magnetic hyperthermia
MNPs	Magnetic nanoparticles
MNPs-MH	Magnetic nanoparticles with magnetic hyperthermia
MNPs-Mel	Melittin-functionalized magnetic nanoparticles
MNPs-Mel-MH	Melittin-functionalized magnetic nanoparticles with magnetic hyperthermia
SAR	Specific absorption rate
TEM	Transmission electron microscopy
XRD	X-ray diffraction
