# Supplementary material for: In Vitro Antitumor Effects of Melittin Attached to Fe3O4 Magnetic Nanoparticles with Synergistic Contribution of Magnetic Hyperthermia
Source: Molecules. 2026 Jun 20;31(12):2171. doi: 10.3390/molecules31122171 (PMC13305969; doi:10.3390/molecules31122171)
Supplement: Supplementary file 1 [file molecules-31-02171-s001.zip › molecules-4336902-supplementary.pdf]

In Vitro Antitumor Effects of Melittin Attached to Fe_3O_4 Magnetic Nanoparticles with Synergistic Contribution of Magnetic Hyperthermia

Alex Câmpian [1], Ioana Bâldea [2], Mara Muntean [1], Cristian Iacoviță [3,*] and Adrian Florea [1]

[1] Department of Cell and Molecular Biology, Faculty of Medicine, "Iuliu Hațieganu" University of Medicine and Pharmacy, 6 Louis Pasteur St., 400349 Cluj-Napoca, Romania;

[2] Department of Physiology, Faculty of Medicine, "Iuliu Hațieganu" University of Medicine and Pharmacy, 1–3 Clinicilor St., 400012 Cluj-Napoca, Romania;

[3] Department of Pharmaceutical Physics-Biophysics, Faculty of Pharmacy, "Iuliu Hațieganu" University of Medicine and Pharmacy, 6 Louis Pasteur St., 400349 Cluj-Napoca, Romania;

* Correspondence: cristian.iacovita@umfcluj.ro

Supplementary Materials

SI1. Magnetic hyperthermia

The specific absorption rate (SAR) is defined as the heat released from a suspension of magnetic nanoparticles (MNPs) in unit time reported to the mass of MNPs. It was used to quantify the heat performance of MNPs. For reliable determination of SAR, the temperature change ΔT versus time curves - where $\Delta T = T_{(t)} - T_0$; $T_{(t)}$ is the temperature at time t and T_0 = 37 °C -, have been fitted with the Box-Lucas equation:

$$\Delta T = \frac{S_m}{k}\left(1 - e^{-k(t-t_0)}\right) \qquad (1)$$

where the fitting parameters S_m and k are the initial slope of the heating curve and the constant describing the cooling rate, respectively. Thus, SAR can be calculated as:

$$\mathrm{SAR} = \frac{c\ m\, S_m}{m_{MNPs}} \qquad (2)$$

where c is the specific heat of the colloid (in our case was approximated with the specific heat of water: $c = 4186\ J/kgK$, the MNPs contribution to the specific heat being negligible); $m = \rho V$ is the mass of colloid, taken as the product between the density ($\rho_{water} = 0,997\ g/cm^3$ for water at 298K) and the volume (0.5 mL); and m_{MNPS} is the mass of MNPs of 0.5 mg. Prior to each measurement the samples have been sonicated for 10 seconds to assure a good colloidal dispersion over the entire aqueous volume. Each SAR value is a mean of three measurements realized on three different samples.

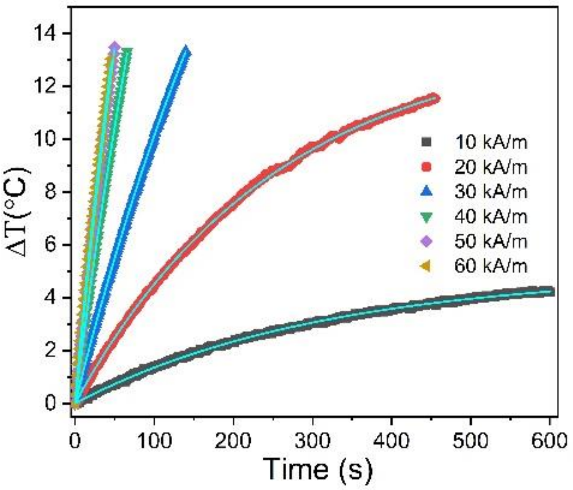

Figure SI1. Typical temperature change ΔT versus time curves fitted with Box-Lucas equation (blue curves) of MNPs dispersed in water at concentration of 0.5 mg/mL, recorded as a function of H (10 – 60 kA/m) at frequency of 355 kHz.

SI2. Melittin calibration curve

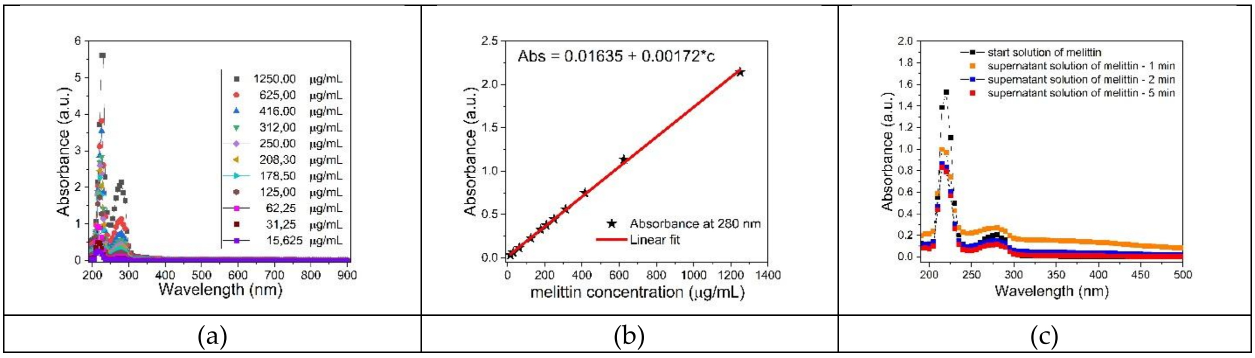

(a)	(b)	(c)

Figure SI2. (a) UV-Vis absorption spectra of aqueous melittin solutions at varying concentrations (1250 to 15,625 µg/mL). (b) Calibration curve showing the relationship between absorbance at 280 nm and melittin concentration (ranging from 1.250 to 15.625 µg/mL) in water, used to determine melittin concentration in subsequent experiments. The red line indicates the linear fit of the experimental data. (c) UV-Vis spectra of aqueous melittin solutions and supernatant solutions collected at different time intervals following the magnetic separation of melittin-coated magnetic nanoparticles from the solution.

SI3. Testing of magnetic hyperthermia for cellular effects

The Caco-2 cells were exposed to MNPs-Mel and different AMF intensities, to select an intensity complying with our conditions to have toxicity effect (viability decrease below 70% of control), yet sufficient cell surviving population to assess the type of cell death and ultrastructural changes. This intensity was 25 kA/m.

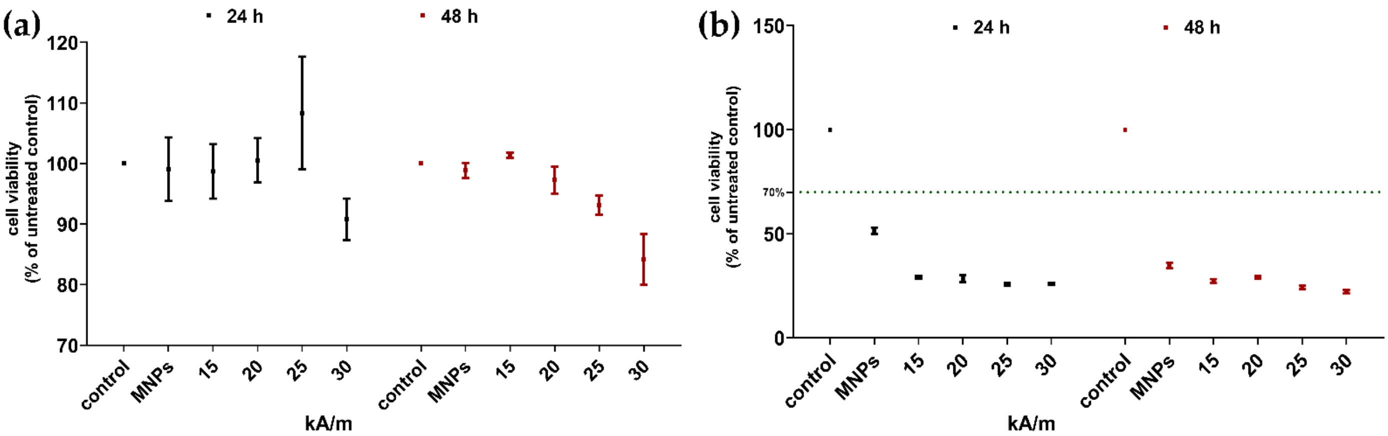

Figure SI3. Viability of Caco-2 cells incubated with 50 µg/mL MNPs (a) and MNPs-Mel (b) and exposed to different MH conditions (n = 3).

SI4. TEM results of BJ and Caco-2 cells incubated with Mel and MNPs-Mel for 72 hours and MH exposure

The BJ cells incubated for 72 h with 2.5 µg/mL Mel presented moderately expanded endoplasmic reticulum, and numerous autophagosomes, while mitochondria preserved normal ultrastructure (Figure SI4a). In the Caco-2 cells incubated for 72 h with 2.5 µg/mL Mel vesicles of small sizes were noted and rare mitochondria with electron-lucent matrix (Figure SI4b). BJ cells incubated with 50 µg/mL MNPs-Mel had altered lysosomes, polymorphous mitochondria, expanded endoplasmic reticulum and moderately increased number of autophagosomes (Figure SI4c). Caco-2 cells incubated with 50 µg/mL MNPs-Mel contained mitochondria with altered cristae with abnormal shapes, large autophagosomes and heterogeneous lipid droplets with dense peripheral region (Figure SI4d).

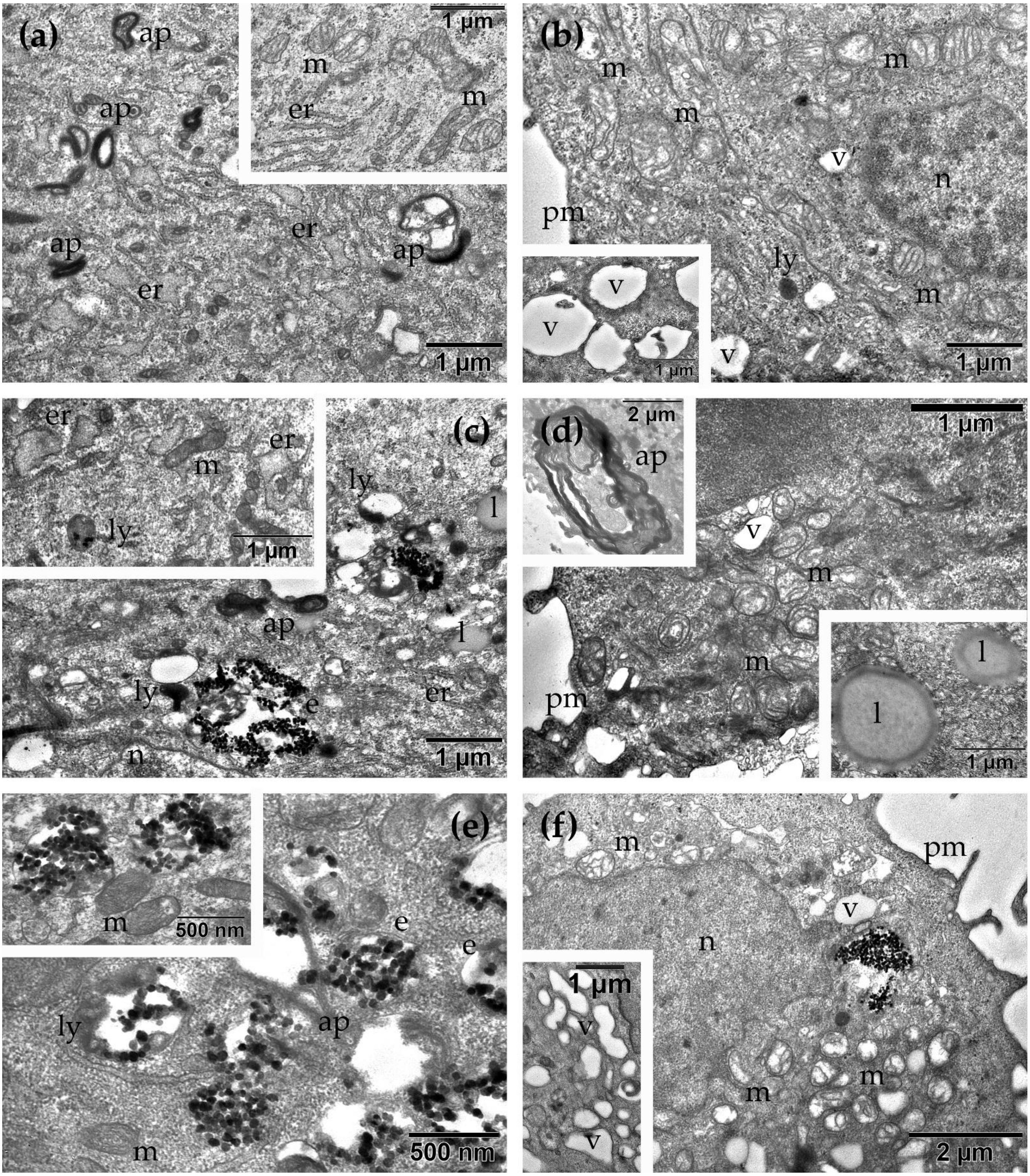

Figure SI4. Relevant TEM images of BJ and Caco-2 cells recorded consecutive to different experimental treatments of 72 h. BJ cells (a) and Caco-2 cells (b) incubated with 2.5 µg/mL Mel (b); BJ cells (c) and Caco-2 cells (d) incubated with 50 µg/mL MNPs-Mel; BJ cells (e) and Caco-2 cells (f) incubated with 50 µg/mL MNPs-Mel and exposed to MH (25 kA/m), after an additional 24 h interval post MH exposure. ap: autophagosome; e: endosome; er: endoplasmic reticulum; l: lipid droplet; ly: lysosome; m: mitochondrion; n: nucleus; pm: plasma membrane; v: vesicle.

Consecutive to MH, in the BJ cells incubated with 50 µg/mL MNPs-Mel many highly altered lysosomes containing MNPs were found. Also, endosomes and lysosomes filled with MNPs had partially disrupted membranes (Figure SI4e). In Caco-2 cells incubated with 50 µg/mL MNPs-Mel and exposed to MH all distinguishable mitochondria showed

electron-lucent matrix and disorganized cristae, along with extensive cellular vacuolation (Figure SI4f).
